# CDKL5 deficiency results in atypical subregion-specific expression of perineuronal nets during mouse visual critical period

**DOI:** 10.3389/fnins.2026.1769195

**Published:** 2026-05-20

**Authors:** Brett Addison Emery, Matthew Everett, Logan Reid Dunn, Billy You Bun Lau, Keerthi Krishnan

**Affiliations:** 1 Department of Biochemistry and Molecular and Cellular Biology, University of Tennessee at Knoxville, Knoxville, TN, United States; 2 Department of Psychology & Neuroscience, University of Tennessee at Knoxville, Knoxville, TN, United States

**Keywords:** CDKL5 null male mice, PNNs expression, rostral V1, V1b, V1M, visual cortex development

## Abstract

Perineuronal nets (PNNs) in the primary visual cortex (V1) are specialized extracellular matrix structures that form predominantly on parvalbumin+ GABAergic neurons, marking the closure of visual critical period plasticity. More recently, PNNs are used to characterize deficits in critical period plasticity in mouse models for neurodevelopmental disorders such as Rett syndrome, Fragile X syndrome, and CDKL5 deficiency disorder. Within the mouse V1, studies typically focus on the expression and function of PNNs within the binocular zone, though PNNs are expressed in other subregions of the V1. The expression and role of these PNNs in other subregions are unknown. Here, we performed a systematic whole V1 characterization of PNN expression using *Wisteria floribunda* agglutinin (WFA) staining, with hemisphere-, subregion-, and anatomical axes- specificity, using a null male mouse model for CDKL5 deficiency disorder during the visual critical period. Patients with CDKL5 deficiency disorder often exhibit cerebral cortical visual impairment, though the underlying mechanisms are unclear. Compared to wild-type controls, *Cdkl5*-null males show regional-specific changes in WFA expression; specifically, decreased all-PNNs in V1M and increased high-intensity PNNs in V1B at P30, and increased WFA pixel intensities in all three V1 subregions at P15, suggesting precocious altered PNN expression in the *Cdkl5*-null V1. In both genotypes, the binocular zone has significantly higher density of PNNs at both ages, compared to the monocular zone and the rostral V1. These results lay the groundwork to probe the roles for PNNs beyond the binocular zone and cumulatively suggest that, during visual critical period, subregion-specific variations in PNN expression may lead to functional consequences within the *Cdkl5*-null cortex.

## Introduction

**Figure 1:**
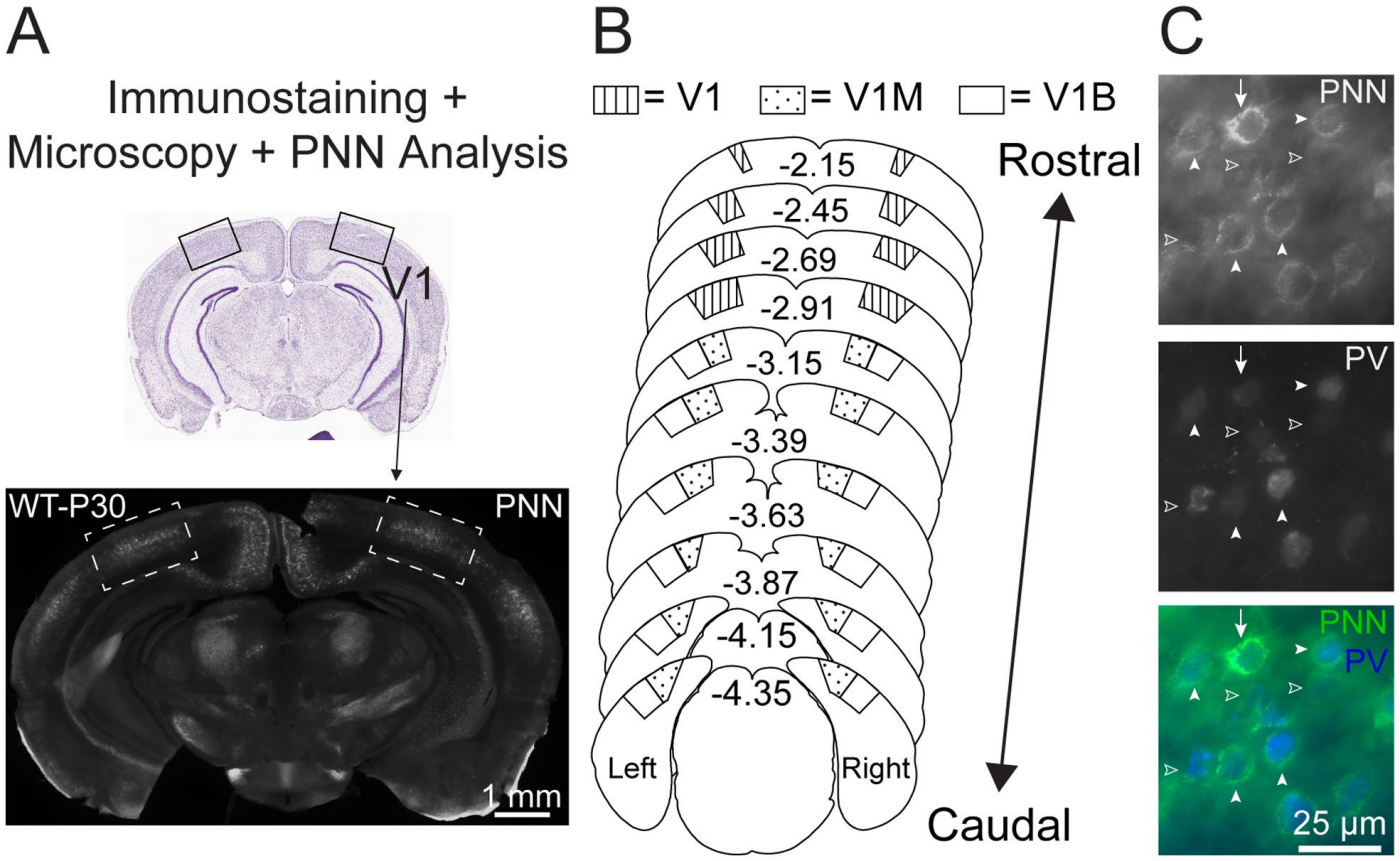
PNN expression analysis across the entire primary visual cortex. **(A)** Top – Nissl-stained coronal mouse brain section, highlighting rostral primary visual cortex (V1; boxed). Bottom: representative coronal wild-type (WT) section immunostained for *Wisteria floribunda* agglutinin (WFA) to identify PNNs (white) in V1 (boxed). In this study, we analyzed postnatal day (P) 15 and P30 brains of WT and CDKL5-null (not shown here). **(B)** Schema of coronal mouse brain sections, depicting rostral to caudal visual cortical subregions analyzed for this study (V1 = rostral V1, V1M = monocular V1, V1B = binocular V1). Values indicate Bregma coordinates according to Paxinos and Franklin (2013). **(C)** Representative epifluorescent images showing PNNs structures (top; bottom-green) colocalize with parvalbumin+ neurons (PV, middle; bottom-blue) in V1 of P30 WT. Arrow indicates high-intensity PNN. Arrowheads indicate lower intensity PNNs. Open arrowheads indicate diffuse immature WFA signal. All types of PNNs surround PV neurons.

Cortical circuits are shaped by experience during sensitive time windows of early postnatal life. The postnatal maturation of cortical interneurons, specifically parvalbumin+ GABAergic neurons, is critical for controlling the timing of critical period plasticity (Hensch, 2005; Hensch et al., 1998; Huang et al., 1999; Wang et al., 2010). This experience-dependent process is concomitant with the expression of specialized extracellular matrix structures called perineuronal nets (PNNs) on parvalbumin+ GABAergic neurons (Begum and Sng, 2017; Celio, 1993; Gundelfinger et al., 2010; Hartig et al., 1992; Hou et al., 2017; Kosaka and Heizmann, 1989; Krishnan et al., 2015; Miyata and Kitagawa, 2017; Nakagawa et al., 1986; Orlando et al., 2012; Sigal et al., 2019; Sorg et al., 2016; Ueno et al., 2018; Ye and Miao, 2013; Sakata et al., 2025; Chavez et al., 2026). PNNs are mainly composed of lecticans, chondroitin sulfate proteoglycans, hyaluronan glycosaminoglycan chains, and other secreted extracellular matrix glycoproteins (Bignami et al., 1992; Carulli et al., 2007; Kwok et al., 2010; Miyata and Kitagawa, 2017). *Wisteria floribunda* agglutinin (WFA) is commonly used as a marker to detect PNNs in the cortex and other brain regions (Brückner et al., 1996; Hartig et al., 1992). WFA specifically binds to N-acetyl galactosamine found on most chondroitin sulfate side chains. Mature PNNs are thought to modulate experience-dependent plasticity (Levelt and Hübener, 2012; Sorg et al., 2016; Takesian and Hensch, 2013; Wingert and Sorg, 2021), as their increase in developing binocular zone of the primary visual cortex correlates with the termination of the critical period, and PNN removal in adult binocular visual cortex restores plasticity, as measured by ocular dominance plasticity assays (Bavelier et al., 2010; Pizzorusso et al., 2006; Pizzorusso et al., 2002). In addition to the binocular zone of the primary visual cortex, PNNs are also expressed in other subregions of the primary visual cortex (Figure 1). Currently, their expression during the critical period is unknown.

**Figure 2:**
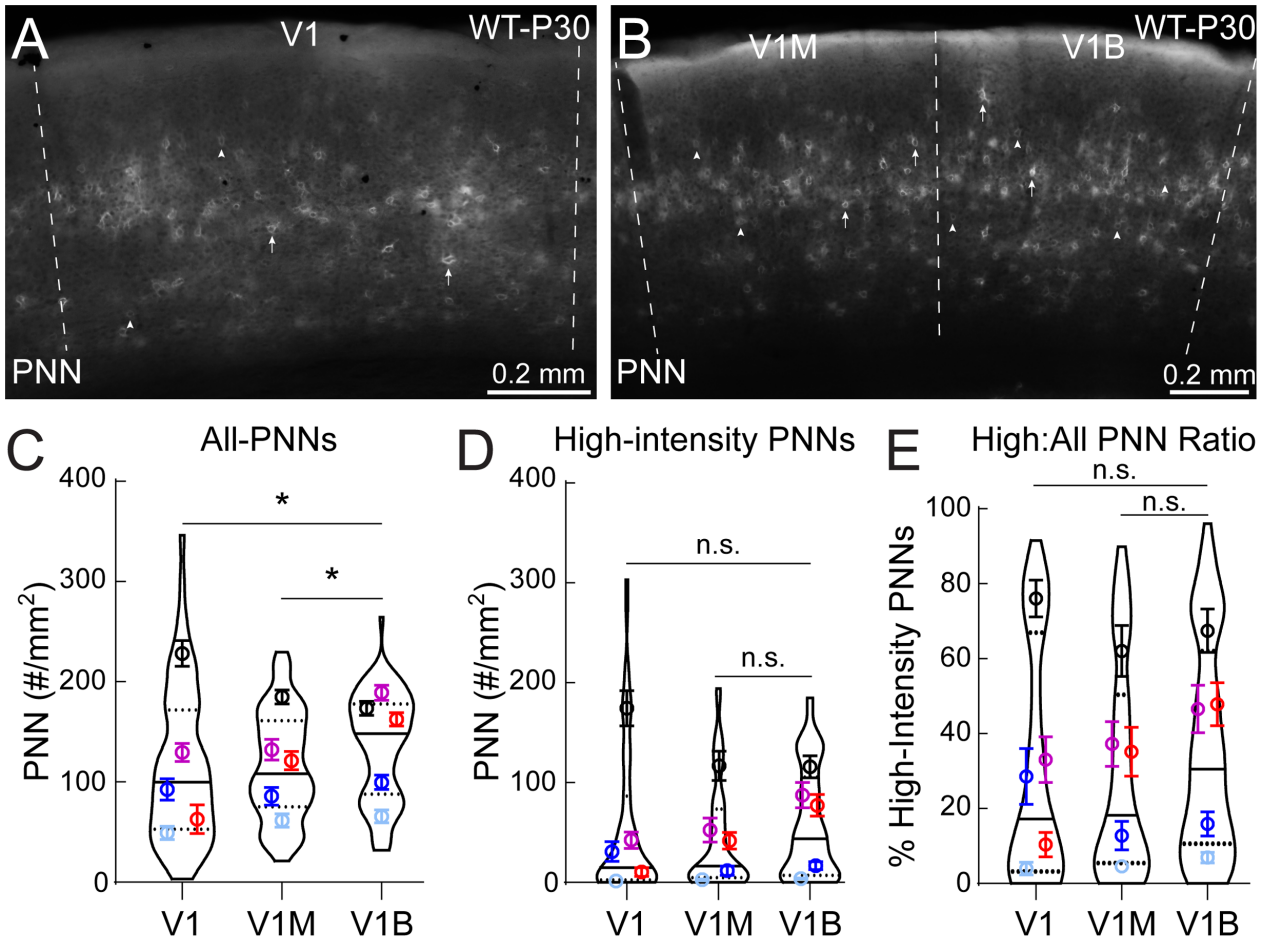
Binocular zone of V1 exhibits higher PNN density than other subregions at P30. **(A, B)** Representative epifluorescent images of PNN expression in rostral V1 (V1, **A**), monocular (V1M) and binocular (V1B) zones **(B)**. Arrowheads and arrows indicate lower intensity and high-intensity PNN structures, respectively. **(C)** V1B exhibited significantly higher all-PNN density, compared to V1M and V1 (Kruskal-Wallis followed by Dunn’s test: *p^*V1B vs V1M*^ = 0.037, *p^*V1B vs V1*^ = 0.013, *p*^*V1 vs V1M*^ = 0.62). **(D)** No significant (n.s.) differences were observed in high-intensity PNNs between the subregions (Kruskal-Wallis followed by Dunn’s test: *p*^*V1 vs V1M*^ = 0.075, *p*^*V1 vs V1M*^ = 0.094, *p*^*V1 vs V1M*^ = 0.98). **(E)** Statistical analysis of high-intensity density to all-PNN density ratio revealed no significant (n.s.) differences between the subregions (Kruskal-Wallis followed by Dunn’s test: *p*^*V1 vs V1M*^ = 0.12, *p*^*V1 vs V1M*^ = 0.16, *p*^*V1 vs V1M*^ = 0.93). For C-E, V1 (64 images), V1M (76 images), V1B (76 images), 5 animals per subregion. Super plots show median (solid line), 25th and 75th quartiles (dash lines) with maximum and minimum, width of violins represents frequency of data points in each region. Each colored circle + lines represent mean ± S.E.M. for a cohort of animals.

**Figure 3:**
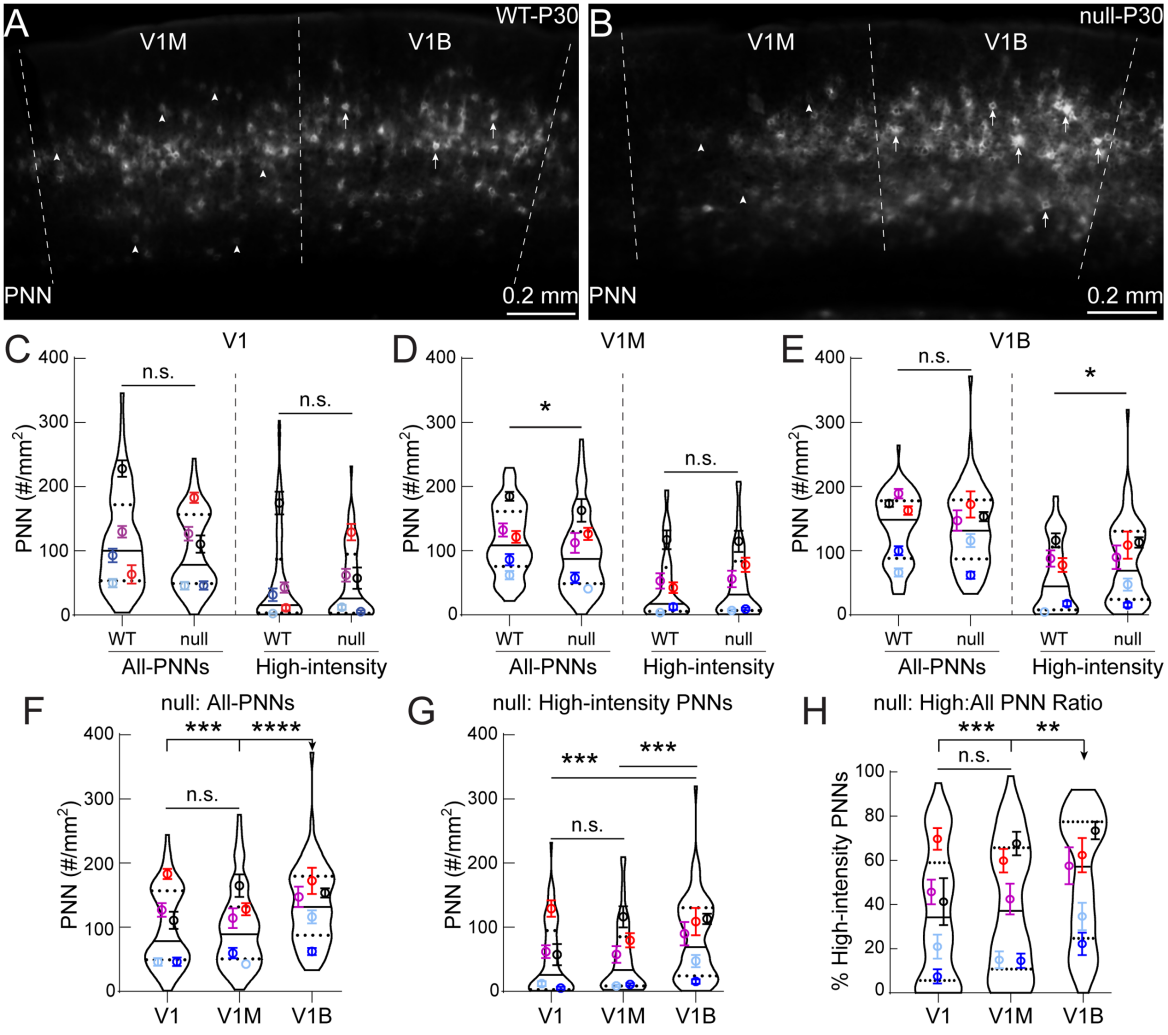
P30 CKLD5-null male mice exhibit abnormal PNN expression in specific subregions of primary visual cortex. **(A, B)** Representative epifluorescent images of PNN expression at P30, showing CDKL5-null (null) **(B)** have fewer PNNs in V1M and more high-intensity PNNs in V1B than WT **(A)**. Arrowheads and arrows indicate lower intensity and high-intensity PNNs, respectively. **(C)** In rostral V1 (V1), statistical analysis revealed PNN densities (All- and high-intensity PNNs) were not significantly (n.s.) different between WT and null (WT: *n =* 64 images; null: *n =* 62 images; 5 animals per subregion; *Mann–Whitney test*: All-PNNs - *p =*  0.26, High-intensity - *p =* 0.88). **(D)** However, in V1M, null showed significantly decreased density for all-PNNs, with no significant change in high-intensity PNNs, compared to WT (*Mann–Whitney test*: All-PNNs - *p = 0.013, High-intensity - *p =* 0.48). **(E)** In V1B, null did not show significant change in all-PNN density, but expressed significantly more high-intensity PNNs compared to WT (*Mann–Whitney test*: All-PNNs - *p =* 0.63, High-intensity - *p = 0.019). **(F, G)** Statistical analysis revealed P30 null V1B exhibited significantly higher all-PNN density **(F)** and higher high-intensity density **(G)**, compared to V1M and V1 (Kruskal-Wallis followed by Dunn’s test: F - ***p^V1B vs V1^ = 0.0005, ****p^V1B vs V1M^ < 0.0001, *p^V1 vs V1M^ =* 0.75; G - ***p^V1B vs V1^ = 0.0003, ***p^V1B vs V1M^ = 0.0004, *p^V1 vs V1M^ =* 0.83). **(H)** Moreover, statistical analysis revealed P30 null V1B exhibited a significantly higher high-intensity density to all-PNN density ratio, compared to V1 and V1M (Kruskal-Wallis followed by Dunn’s test: ***p^V1B vs V1^ = 0.001, **p^V1B vs V1M^ = 0.005, *p^V1 vs V1M^ =* 0.54). For **(D, E)**, WT: *n =* 76 images, null: *n =* 72 images; 5 animals per subregion. For F–H, V1 (62 images), V1M (73 images), V1B (72 images), 5 animals per subregion. For C–H, n.s. = not significant; super plots: violin plots show median (solid line), 25th and 75th quartiles (dash lines) with maximum and minimum, width of violins represents frequency of data points in each region. Each colored circle + lines represent mean ± S. E. M. for a cohort of animals.

PNN expression is also used as a marker to detect alterations in sensory processing and/or changes in inhibitory networks in mouse models for neurodevelopmental disorders such as Rett syndrome, Fragile X syndrome, and CDKL5 deficiency disorder (Carstens et al., 2021; Donato et al., 2013; Krishnan et al., 2017; Krishnan et al., 2015; Lau et al., 2020a; Lau et al., 2020b; Patrizi et al., 2020; Pizzo et al., 2016; Reinhard et al., 2019; Wen et al., 2018). CDKL5 deficiency disorder is characterized by seizures, intellectual disability, motor deficits, and social impairments (Brock et al., 2021; Demarest et al., 2019; Siri et al., 2021; Terzic et al., 2021; Zhu and Xiong, 2019). As CDKL5 is on the X chromosome, majority of the children with CDKL5 disorder are girls, though boys are also affected (Bahi-Buisson et al., 2012; Bahi-Buisson and Bienvenu, 2012; Fehr et al., 2016; Fehr et al., 2013; Guerrini and Parrini, 2012; Kalscheuer et al., 2003; Weaving et al., 2004). Patients often exhibit cerebral visual impairment (Brock et al., 2021; Mari et al., 2005; Quintiliani et al., 2021), though the underlying mechanisms are unclear. Mouse models of the CDKL5 deficiency disorder recapitulate some of these symptoms, with persistent reduction in response amplitude, reduced visual acuity, and defective contrast function (Mazziotti et al., 2017). Interestingly, these deficits in visually evoked responses are observed in young adult mice (~P60-80) and at the peak of visual critical period plasticity around postnatal day 28 (~P28), but not before (~P25). These results correlate with increased PNN expression within the binocular zone of the *Cdkl5*-null males at ~P35 (Pizzo et al., 2016), suggesting that the timing of critical period plasticity and the proper wiring of visual cortex circuitry are likely affected in rodent models for CDKL5 deficiency disorder. In this study, we used *Cdkl5*-null male mice, which carry a complete loss of CDKL5 protein function and provide the most robust and penetrant phenotypes. Heterozygous females, which more closely model the human condition, exhibit mosaic CDKL5 expression due to random X-inactivation, resulting in highly variable and milder phenotypes that require substantially larger cohorts for reliable detection (Fuchs et al., 2018; Terzic et al., 2021). Given the subtle PNN phenotypes observed even in null males (see “Discussion”), characterization in the null male model was a necessary first step before extending to the more variable female model.

Though the role of cortical PNNs was established by pivotal studies in the mouse primary visual cortex, all of these studies cited above, performed immunostaining with representative brain sections and mainly focused on the binocular zone (V1B). A systematic characterization of PNNs in the whole primary visual cortex during critical period is crucial to determine the role of PNNs in establishing and/or maintaining visual cortex function. This characterization is also critical to interpret other functional analysis such as visually evoked potentials, which are not typically restricted only to the binocular zone and are relevant as tools for atypical cortical activity in neurodevelopmental disorders (Demarest et al., 2019; LeBlanc et al., 2015; Mazziotti et al., 2017; Wang et al., 2012). Thus, we performed a systematic characterization of PNN expression and density across the developing visual cortex of wild-type (WT) and *Cdkl5*-null (null) mice during visual critical period. We analyzed three PNN populations: (1) high-intensity (mature) PNNs, (2) high-intensity and dimmer expressing PNNs (all-PNNs) and (3) PNN pixels (histogram). At P30, manual counting of high-intensity PNNs shows no differences across the 3 subregions of the WT visual cortex (V1, V1M (monocular zone) and V1B). In contrast, null mice show a significant increased high-intensity PNN density in V1B when compared to V1 and V1M. Analysis of all-PNNs in WT reveals a significant increase in V1B density compared to V1 and V1M, which is also observed in null mice. Across genotype, we find null mice have significantly reduced all-PNNs density in V1M and increased high-intensity PNNs density in V1B, compared to WT mice. At P15, histogram PNN intensity analysis reveals significant increases in WFA intensity in null mice, in all 3 subregions, when compared to WT mice. Together, these results show a precocious increase in PNN expression in the *Cdkl5*-null primary visual cortex during the critical period. These results suggest that appropriate expression of CDKL5 is critical for neuronal plasticity during early development, setting the stage for studying PNN expression and function over age, with subregion specificity in the mouse primary visual cortex.

## Materials and methods

### Animals

We used the following mouse strains from The Jackson Laboratory: *Cdkl5* hemizygous [B6.129 (FVB) - Cdkl5^tm1.1Joez^/J; null] (Wang et al., 2012) and wild type (WT) (C57BL/6 J). Young male mice (P15, *n =* 3 animals per genotype; P30, *n =* 5 per genotype) were maintained on a 12-h light–dark cycle (lights turned on at 07:00 a.m.) and received food ad libitum. Procedures were conducted in accordance with the National Institutes of Health’s Guide for the Care and Use of Laboratory Animals. Protocols were approved by the University of Tennessee in Knoxville’s Institutional Animal Care and Use Committee.

### Immunohistochemistry

WT and null male mice were anesthetized with isoflurane and perfused with 1X phosphate buffer saline (PBS) solution followed by 4% paraformaldehyde (PFA) dissolved in PBS. Brains were extracted and post-fixed in PFA overnight at 4 °C. Prior to sectioning, brains were treated with 30% sucrose in PBS overnight at room temperature. A cut was made on the left ventral-medial brain hemisphere to denote orientation during subsequent tissue processing and analysis. A freezing microtome was used to cut coronal brain sections at 70.0 μm. Sectioning and subsequent histological and imaging steps were done in cohorts, consisting of one WT and one null, to minimize technical variations. Immunohistochemistry was performed as previously described (Krishnan et al., 2015). Briefly, free-floating brain sections were blocked in 10% normal goat serum (NGS) and 0.5% Triton-X in PBS for 3 h. Then, sections were incubated overnight with biotin conjugated Lectin from *Wisteria floribunda* agglutinin (L1516, Sigma-Aldrich) (1:500) in a 5% NGS and 0.25% Triton-X in PBS. Some tissues were also co-incubated with PV antibodies (mouse, 1:1,000) (P3088, Sigma-Aldrich). The next day, sections were incubated for 4 h with Streptavidin AlexaFluor-488 (S32354) and anti-mouse CY5 (A10524) (ThermoFisher Scientific; 1:1,000) where appropriate in a 5% NGS and 0.25% Triton-X in PBS. Sections were counterstained with the DAPI (1:1000) for 5 min and mounted on slides in Fluoromount-G for imaging.

### Image acquisition

Single-plane PNN images of the entire visual cortex from both hemispheres of each brain section were acquired using an epifluorescence microscope (Keyence BZ-X710; Keyence Corp.) equipped with a 10X objective and motorized stage. Imaging settings were determined for each cohort of animals based on the null to minimize overexposure, as preliminary observations suggested brighter PNN expression in null tissues. One cohort consisted of one null and one age-matched wild-type control. Exposure settings for each cohort were determined as previously described (Lau et al., 2020b). Briefly, we identified the exposure time that gives rise to the fewest number of saturated pixels within frame for each tissue. Then, we decreased the exposure time by 1 unit. We completed this process for all null tissues within the cohort and averaged them to get a final exposure time and used this averaged time for final image acquisition of both wild-type and null tissues within the cohort. Images were stitched using the Keyence BZ-X Analyzer software.

### Quantitative image analysis

PNN analysis for each hemisphere was performed in ImageJ. We analyzed 10–15 coronal brain sections (both hemispheres) per brain. Rostral V1 (V1), binocular zone (V1B), and monocular zone (V1M) of each image were identified and outlined by overlaying maps from the Paxinos and Franklin’s mouse brain atlas (Paxinos and Franklin, 2013).

PNN expression exists along a maturation continuum: WFA staining around individual neurons ranges from diffuse labeling of immature extracellular matrix to strong, well-defined net-like structures surrounding the soma and proximal dendrites of mature PNNs. High-intensity PNNs, characterized by perisomatic localization, are the most widely quantified class in the field and are thought to represent functionally mature structures that actively constrain synaptic plasticity. To capture PNN expression more comprehensively, we also quantified “all-PNNs,” which include both high-intensity (mature) and dimmer (weak/diffuse) PNNs. This dual quantification approach allows us to distinguish between changes in overall mature PNN density and shifts in PNN maturation state, which may reflect different aspects of inhibitory network development. Additionally, we performed pixel-intensity-based analysis to capture the full range of WFA signal, including diffuse extracellular matrix labeling that has not yet coalesced into identifiable PNN structures. This approach is particularly informative at early developmental stages (P15), when classic PNN morphology is minimal in the visual cortex. However, pixel-based analysis does not account for PNN size, neuronal soma size, or the spatial extent of individual PNN structures; thus, this measure is complementary to, rather than a replacement for, manual PNN counting.

High-intensity PNNs, representing the most mature PNNs, were counted as previously described (Lau et al., 2020b). Briefly, the contrast setting from ImageJ was maximized to remove weaker signals from the images. The remaining signals were manually quantified as a mature PNN if it retained 80% of its original shape (before contrast adjustment). To determine 80% for each high-intensity PNN, we used the maximized contrast image and the segmented line tool to measure the perimeter of the partial PNN. In the original image, the tips of the segmented line were then connected with a new segmented line to fully encompass the whole PNN. Finally, percentage was calculated by dividing the perimeter value obtained from the partial PNN by the total perimeter (sum of the 2 segmented lines). Detailed protocol and video for mapping and high-intensity PNN analysis can be found in Protocols.^1^

**Figure 4:**
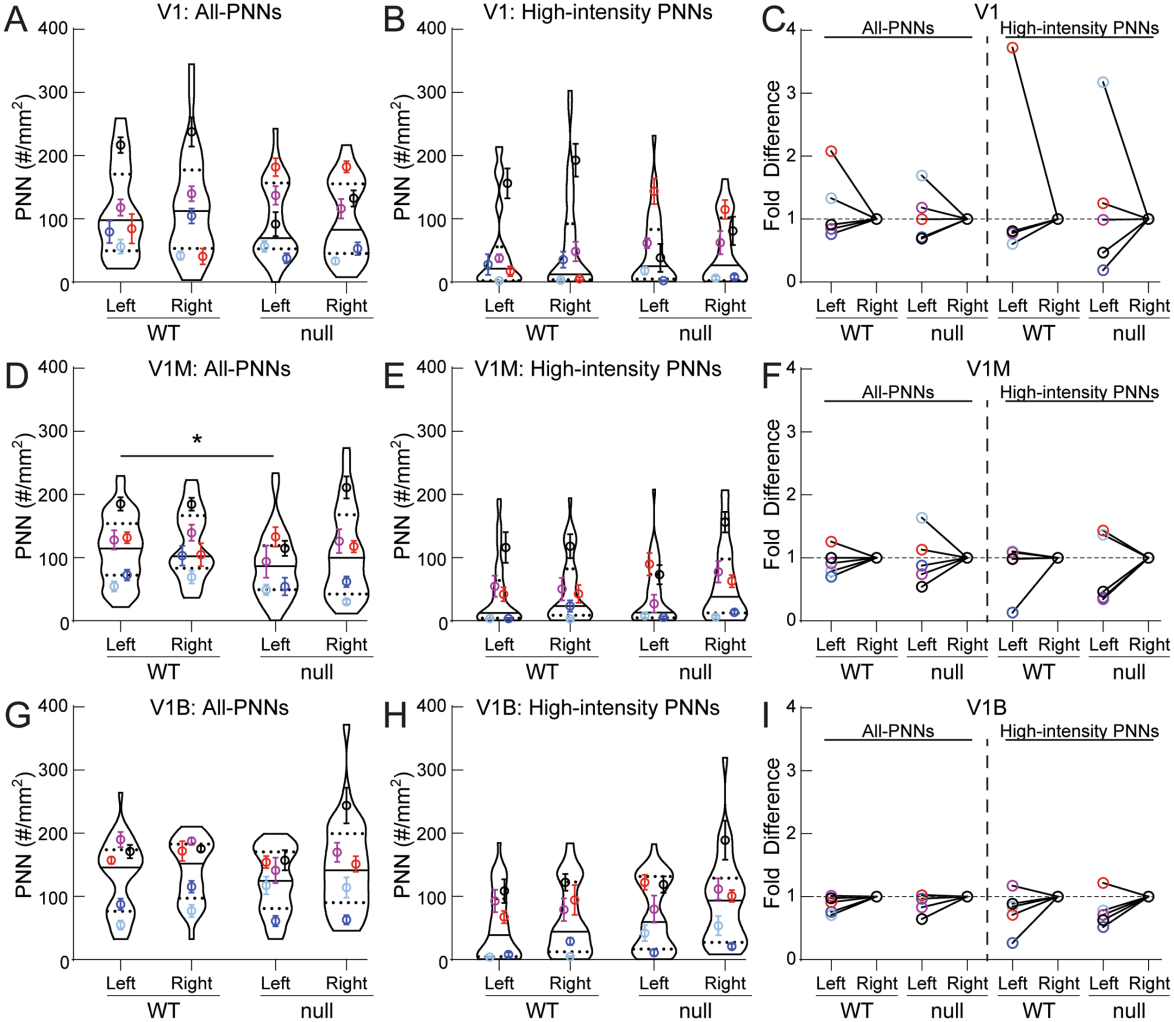
Individual brains exhibit similar PNN density between hemispheres in all primary visual cortical subregions at P30. On average and within genotype, WT and null exhibited similar all-PNN density **(A, D, G)** and high-intensity PNN density (B, E, H) between left and right hemispheres of rostral V1 **(A, B)**, V1M **(D, E)** and V1B **(G, H)**. Overall, there were no hemisphere-specific changes between WT and null visual cortex, except for V1M, where null showed a significant reduction in all-PNN density in the left hemisphere **(D)** (Kruskal-Wallis followed by Dunn’s test: V1M: *p^WT-left vs null-left^ = 0.035). **(C, F, I)** Panels displaying fold difference values (left/right hemisphere PNN density) for individual brains to transparently illustrate inter-animal biological variability in hemisphere asymmetry. **(A, B, D, E, G, H)** Super plots: violin plots showing median (solid line), 25th and 75th quartiles (dash lines) with maximum and minimum, width of violins represents frequency of data points in each region. Each colored circle + lines represent mean ± S.E.M. for a cohort of animals. *N* = 31-42 images, 5 animals per group. See Table 1 for full statistical results.

All-PNNs (including high-intensity PNNs) were manually quantified from the “original” image (no contrast nor brightness adjustment); all-PNNs had 100% complete borders. To determine left–right asymmetry of PNN expression (Figure 4), for each brain, we averaged the PNN densities (all-PNNs or high-intensity PNNs) from the left hemisphere and averaged the PNN densities from the right hemisphere. Then, we divided the averaged PNN density value of the left hemisphere by the averaged PNN density value of the right hemisphere to determine the level of PNN asymmetry.

**Table 1:** Statistical *p*-values for Figure 4.

	Figure 4A All	Figure 4B High	Figure 4D All	Figure 4E High	Figure 4G All	Figure 4H High
WT: Left vs. Right	0.96	0.94	0.60	0.35	0.32	0.30
null: Left vs. Right	1.00	0.93	0.28	0.090	0.20	0.20
Left: WT vs. null	0.40	0.92	0.035*	0.92	0.59	0.11
Right: WT vs. null	0.43	0.91	0.16	0.39	0.85	0.09

*p < 0.05.

**Figure 5:**
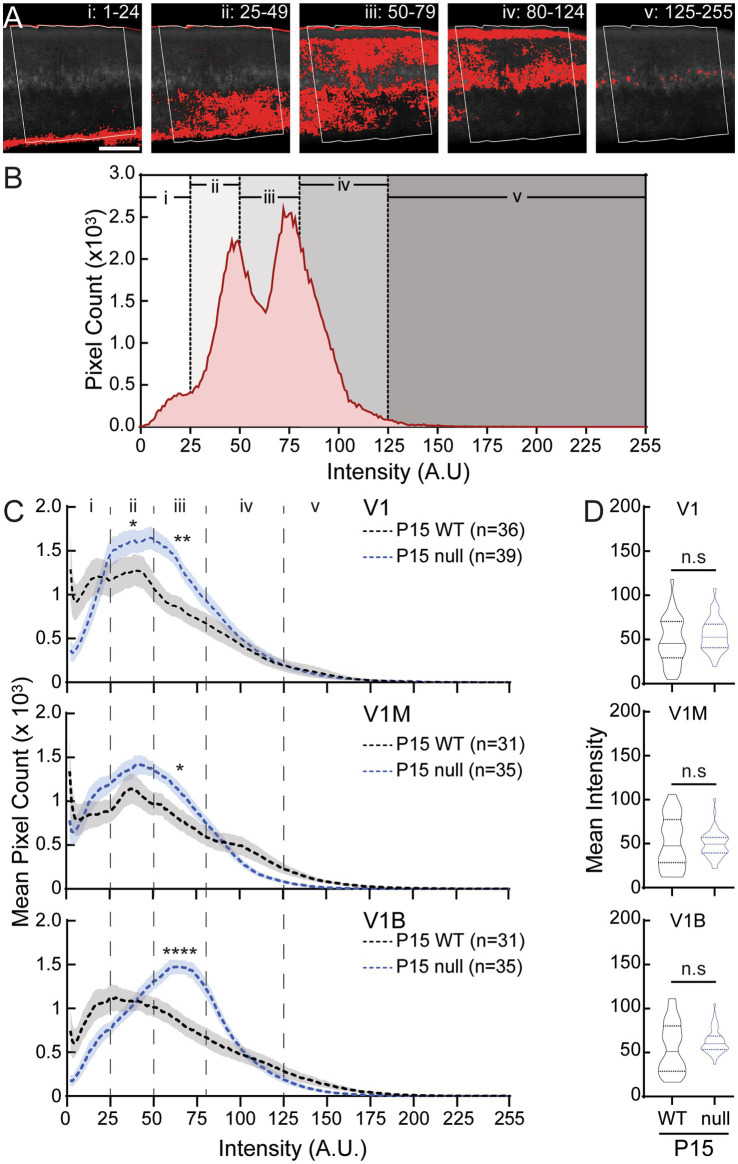
CDKL5-null male mice express increased WFA intensity in all subregions of the primary visual cortex at P15. **(A)** One representative greyscale WFA image in V1B (boxed) is used as an example to show five different ranges of pixel intensity in ImageJ, and their locations in different layers (i–v). Red indicate signals measured at each of the five intensity ranges. **(B)** Histogram showing intensity distribution of all WFA signals from the same image in **(A)**. **(C)** Averaged histograms showing WFA intensity distribution in V1 (top), V1M (middle) and V1B (bottom) of WT (black) and null (blue) (dash lines). In V1, null had significantly higher WFA intensities in the ranges of ii and iii compared to WT (*Mann–Whitney test*: ranges i, *p =* 0.81; ii, *p = 0.019; iii, **p = 0.0019; iv, *p =* 0.11; v, *p =* 0.44). In V1M, null had significantly higher WFA intensities in the range of iii compared to WT (*Mann–Whitney test*: ranges i, *p =* 0.32; ii, *p =* 0.17; iii, *p = 0.050; iv, *p =* 0.48; v, *p =* 0.94). In V1B, null had significantly higher WFA intensity in iii compared to WT (*Mann–Whitney test*: ranges i, *p =* 0.11; ii, *p =* 0.77; iii, ****p < 0.0001; iv, *p =* 0.14; v, *p =* 0.53). Mean (dash lines) ± S. E. M. (shades) are shown. *N* values represent numbers of images analyzed for each condition, with 3 animals per group. **(D)** Combined-hemisphere statistical analysis of V1 (top), V1M (middle) and V1B (bottom) revealed no significant (n.s.) differences in mean WFA intensity without the different groupings between WT and null (*Mann–Whitney test*: *p^V1-WT vs V1-null^ =* 0.17, *p^V1M-WT vs V1M-null^ =* 0.99*, p^V1B-WT vs V1B-null^ =* 0.19). Violin plots showing median (solid line), 25th and 75th quartiles (dash lines) with maximum and minimum, width of violins represents frequency of data points in each region. *N* values for each group are the same as in **(C)**.

**Figure 6:**
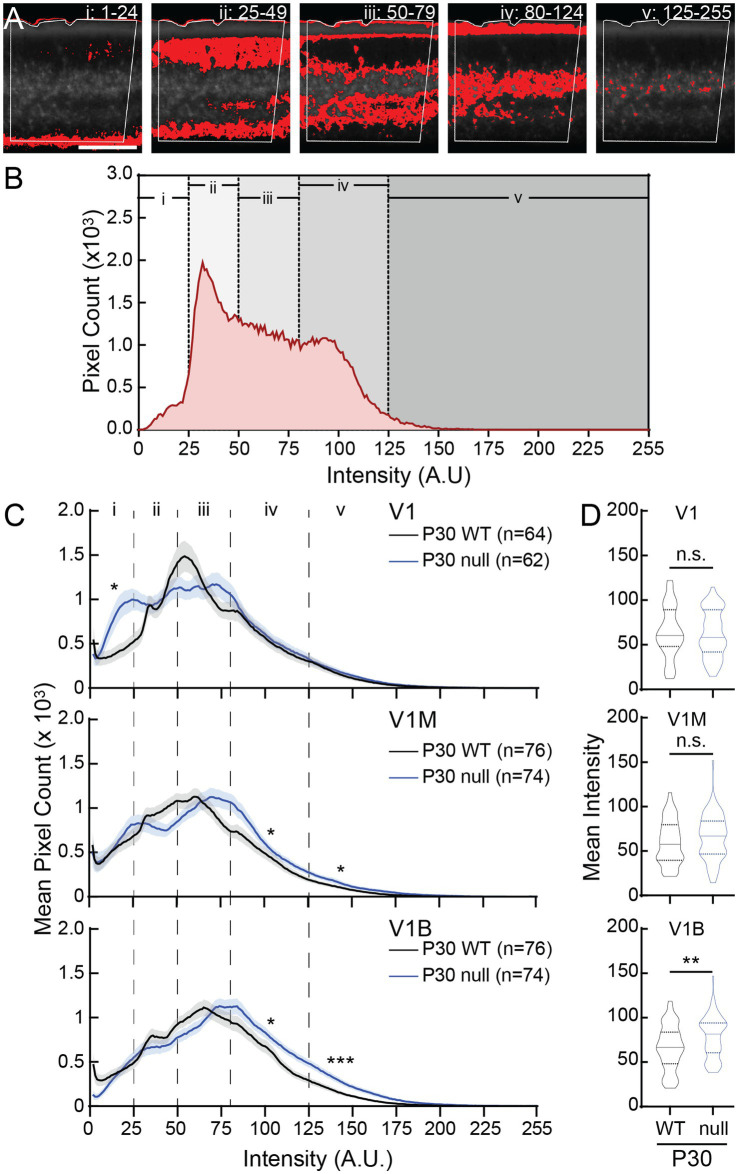
CDKL5-null male mice express increased WFA intensity in a V1 subregion-specific manner at P30. **(A)** One representative greyscale WFA image in V1B (boxed) is used as an example to show five different ranges of pixel intensity in ImageJ and their locations in different layers (i–v). Red indicate signals measured at each of the five intensity ranges. **(B)** Histogram showing intensity distribution of all WFA signals from the same image in **(A)**. **(C)** Averaged histograms showing WFA intensity distribution in V1 (top), V1M (middle), and V1B (bottom) of WT (black) and null (blue) at P30 (solid lines). In V1, null had significantly higher WFA intensity in the range of i compared to WT (*Mann–Whitney test*: ranges i, *p = 0.039; ii, *p =* 0.74; iii, *p =* 0.98; iv, *p =* 0.66; v, *p =* 0.23). In V1M, null had significantly higher WFA intensity in the ranges of iv and v compared to WT (*Mann–Whitney test*: ranges i, *p =* 0.94; ii, *p =* 0.17; iii, *p =* 0.64; *iv, *p =* 0.021; v, *p = 0.027). In V1B, null had significantly higher WFA intensity in the ranges of iv and v compared to WT (*Mann–Whitney test*: ranges i, *p =* 0.57; ii, *p =* 0.20; iii, *p =* 0.96; *iv, *p =* 0.011; v, ***p = 0.0002). Mean (solid lines) ± S. E. M. (shades) are shown. *N* values represent number of images analyzed for each condition, with 5 animals per group. **(D)** Combined-hemisphere statistical analysis revealed no significant (n.s.) differences in mean WFA intensity between WT and null in V1 (top) and V1M (middle). However, null expressed significant higher mean WFA intensity in V1B (bottom) (*Mann–Whitney test*: *p^V1-WT vs. V1-null^ =* 0.68, *p^V1M-WT vs. V1M-null^ =* 0.13, **p^V1B-WT vs. V1B-null^ = 0.007). Violin plots showing median (solid line), 25th and 75th quartiles (dash lines) with maximum and minimum, width of violins represents frequency of data points in each region. *N* values for each group are the same as in **(C)**.

For histogram and mean intensity analyses (Figures 5, 6), individual intensity values within each subregion region-of-interest (ROI) were acquired from ImageJ by selecting “Analyze” → “Tools” → “Save XY Coordinates” and analyzed in GraphPad Prism. In Figures 5, 6, we illustrate the different histogram ranges in the PNN image after conversion to grey scale. The ranges of intensity were set under the Threshold option in ImageJ. The conversion of intensity ranges between weighted greyscale images and the green signal histogram was performed using the following equation: grey = 0.587green.

This pixel-intensity approach provides a complementary metric to manual PNN counting, analogous to the “diffuse fluorescence” measure employed in brain-wide PNN atlas studies (Lupori et al., 2023), and was analyzed following principles of standardized WFA intensity quantification (Slaker et al., 2016).

### Statistical analysis

For graphical figures except Figure 4, the analyses were performed using combined hemisphere data. For Figure 4, analyses were performed on parsed left and right hemisphere data. We used Mann–Whitney tests for pair-wise comparison between wild-type and null brains (Figures 3C–E, 5, 6) and Kruskal-Wallis followed by Uncorrected Dunn’s tests for comparisons between more than 2 groups (Figures 2, 3F–H, 4). The threshold for significance was set at 0.05. Where appropriate, the numbers of animals and images are indicated within the figure legends. Statistical analyses were performed in GraphPad Prism and figures were generated in Adobe Illustrator.

## Results

We performed a systematic characterization of PNN expression across the subregions of the primary visual cortex by collecting most coronal brain sections covering the entire visual cortex (Figures 1A,B). The primary visual cortex encompasses ~2.2 mm of the mouse brain [Bregma coordinates −2.15 mm to −4.35 mm (Paxinos and Franklin, 2013)] and is composed of the rostral V1 (V1), monocular zone (V1M), and binocular zone (V1B) subregions (Figure 1B). Within the cortex, PNNs surround the soma and proximal dendrites of parvalbumin+ GABAergic neurons (Agetsuma et al., 2018; Bartos et al., 2002; Cardin et al., 2009; Celio, 1993; Cho et al., 2020; Faini et al., 2018; Hartig et al., 1992; Karunakaran et al., 2016; Ko et al., 2013; Sohal et al., 2009; Ueno et al., 2018); PNN expression is predominantly in superficial layers of the cortex (Layers II/III, IV), with minimal expression in deeper layers (Layers V, VI). As assessed by *Wisteria floribunda* agglutinin (WFA) staining (Brückner et al., 1996; Hartig et al., 1992), PNN expression around individual neurons can be strong, weak or diffused (Figure 1C, arrows, arrowheads or open arrowheads, respectively). These intensity classes are thought to reflect the maturation state of perineuronal net assembly, with strong staining corresponding to fully mature, functionally constraining structures and weaker staining representing earlier stages of extracellular matrix condensation around the neuronal soma (Slaker et al., 2016; Xia et al., 2021).

During the peak of mouse critical period plasticity (P30), WT males show more distinct PNN expression within the V1M and V1B subregions compared to rostral V1 (Figures 2A,B). Through manual quantification of all-PNNs across these subregions, we found that V1B showed significantly higher PNN density compared to V1 and V1M (Figure 2C). However, the density of mature, high-intensity PNNs did not differ between subregions (Figure 2D). The ratio of high-intensity PNNs to all-PNNs (represented as a percentage) did not vary significantly between subregions (Figure 2E), suggesting that although PNN density is highest in V1B, the majority of PNNs are not of full maturity. Together, these results show that PNN expression is subregion-specific in WT males, with V1B showing significantly greater PNN density than V1M or rostral V1 following the peak of critical period plasticity.

Next, we determined if the male mouse model for CDKL5 deficiency disorder displayed subregion-specific alterations in PNN expression, as was previously shown in the V1B (Pizzo et al., 2016). We found PNN expression in P30 null males appeared to be distinctly different in multiple visual cortex subregions compared to WT controls (Figures 3A,B). Compared to WT controls, all-PNN density within V1M was significantly decreased in P30 null males, while no significant difference in high-intensity PNN density was observed between conditions (Figure 3D). In contrast, high-intensity PNN density within V1B was significantly greater in null than WT, with no significant difference in all-PNN density (Figure 3E). We observed no significant differences in PNN densities between conditions in rostral V1 (Figure 3C). While comparing within the null genotype, PNN density in V1B was significantly higher compared to V1M and rostral V1, with higher densities of all-PNNs, high-intensity PNNs and ratio of high-intensity to all-PNN (Figures 3F–H, respectively). These results are in contrast with the WT profile, where only density of all-PNNs was significantly higher in V1B (Figures 2C–E). Together, these results suggest that subtle, yet significant subregion-specific differences exist in PNN density within the *Cdkl5*-null mouse visual cortex at P30.

We previously reported that the adult WT female primary somatosensory cortex exhibited anatomical axis- and hemisphere-specific changes in high-intensity PNN density (Lau et al., 2020b), which may contribute to functional specialization within cortical circuitry. To determine if the developing visual cortex of male mice also exhibits such anatomical and hemisphere-specific expression patterns during critical period, we plotted PNN densities (all-PNNs and high-intensity PNNs) across the rostral-caudal axis (Supplementary Figure 1). In contrast to the adult primary somatosensory cortical PNN density distribution (Lau et al., 2020b), we observed no significant differences in either all- or high-intensity PNN density within genotype along the rostral-caudal axis.

Next, we investigated if there were any hemisphere-specific changes in PNN expression as reported in the adult female brains (Lau et al., 2020b). Fold difference changes within left and right hemispheres of individual brains are shown in Figures 4C,F,I to highlight the individual variation in PNN density across the sub-regions, while the other panels focus on the aggregate statistics and average data. Plotting PNN density of individual sections between the left and right hemispheres, we observed no significant difference in all-PNN (Figures 4A,D,G) or high-intensity PNN (Figures 4B,E,H) density within genotype across all visual cortex subregions. Analyzing left–right asymmetry within individual brains showed remarkable consistency in V1B (Figure 4I), while V1 and V1M showed minor individual variations (Figures 4C,F, red and light blue circles), indicative of similar PNN densities between hemispheres in both grouped and individual analyses. One statistically significant difference was observed between genotypes in the left hemisphere of V1M: all-PNNs (Figure 4D), though the overall distribution remains similar. These findings cumulatively suggest that (*1*) hemisphere laterality has not been established within the primary visual cortex during the peak of critical period plasticity, and (*2*) this process is not dysregulated at P30 within the male *Cdkl5*-null visual cortex.

As we observed an increase in the high-intensity PNN density within V1B of P30 null males, we wondered if the etiology of CDKL5 deficiency disorder was similar to that of the *Mecp2*-null visual cortex, with a precocious increase in high-intensity PNN density, indicative of an accelerated critical period (Krishnan et al., 2015). Thus, we analyzed PNN expression at the start of the critical period, in P15 WT and null brains. WFA staining in developing visual cortex is typically weak and diffuse after eye opening (~P12), with some layer IV neurons displaying a classic net-like structure (Krishnan et al., 2015; Pizzorusso et al., 2002; Ye and Miao, 2013). To capture the WFA staining patterns in this immature state, we performed pixel intensity analysis across individual images using ImageJ. In a representative example, the histogram intensity values (Figures 5A,B) corresponded to varying staining patterns across layers, with (i, ii) intensity value of 1–49 representing fiber staining in corpus callosum and deeper layers of the cortex, (iii) 50–79 representing weak yet specific staining in superficial and deep cortical layers, (iv) 80–124 identifying more defined PNN structures in Layer IV as well as background staining in Layer I, and (v) over 125 identifying high-intensity PNNs, mostly in Layer IV. A binned intensity analysis over all P15 images of WT and null sections showed higher mean intensities in null brains, especially over the weak staining in upper layers (Figure 5C, iii), in all three subregions. There was no statistically significant difference in subregion-specific overall mean intensity measurements between P15 WT and null, though the distributions were distinct (Figure 5D). Together, these results indicate an overall precocious increase in PNN expression in different layers and structures, though not of the typically measured mature high-intensity PNNs.

We performed a similar analysis with P30 brain sections, to relate the histogram analysis to manually quantified PNNs (all- and high-intensity PNNs). As seen with P15 tissues, the intensities between 80 and 124 delineated the most well-formed PNNs in Layer IV (Figures 6A,B, iv), with intensities of 125 and above identifying the high-intensity PNNs (Figures 6A,B, v). In the null rostral V1, we detected a significant increase in the fiber staining in the corpus callosum and deeper layers (i), with no overall change in mean intensity (Figures 6C,D, top panels). In the V1M, pixel intensity analysis showed the alternating dynamics in intensity groups (ii) and (iii) between the WT and null, with significant increases in pixel intensity in (iv) and (v) groups, corresponding to layers I and IV, of the null V1M (Figures 6C,D, middle panels). These results are in contrast with the observed decrease in all-intensity PNN density in the null V1M, from the manual counting of PNN structures (Figure 3D). In the V1B, pixel intensity analysis showed an increase in mean intensity in the null V1B compared to WT controls, recapitulating the manual high-intensity PNN analysis (Figures 6C,D, v, bottom panels vs. Figure 3E). Together, these results reiterate the increase in defined PNN structures in the null V1B, and identify nuance changes at different levels of PNN expression in the V1M.

## Discussion

**Figure 7:**
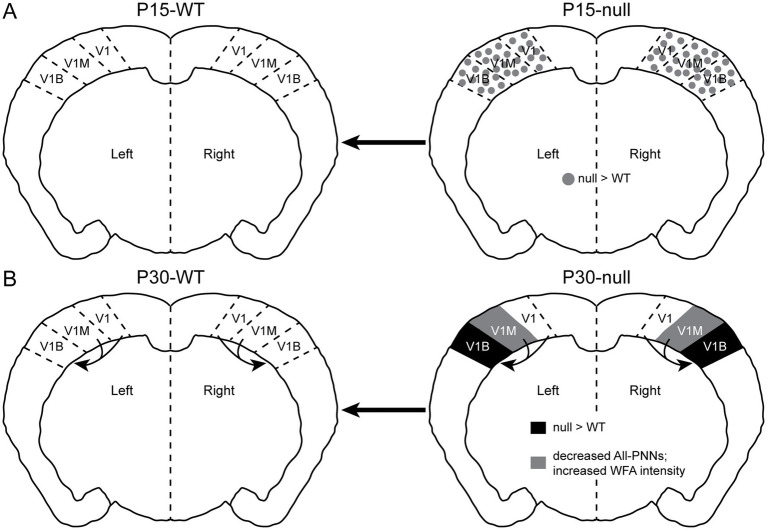
Summary of PNN expression changes between WT and CDKL5-null during critical period in subregions of the primary visual cortex. **(A)** Before the beginning of critical period at P15, null exhibited increased diffuse WFA expression in V1, V1M, and V1B (grey dots), suggesting the beginnings of PNN dysregulation. **(B)** At the peak of critical period, null exhibited increased PNN expression in V1B (black). In V1M, null had nuanced changes in PNN expression compared to WT: null showed a decrease in the density of overall PNN structures, by manual counting (Figure 3D), and a higher intensity of WFA signal (Figure 6C). Together, these results suggest fluid and compensatory changes in PNN expression in null V1M at these ages. The impact of such changes over time, on synaptic plasticity and visual cortex function, particularly in V1M, needs further work.

PNNs are widely expressed in the rodent brain; in particular, the different sensory cortical regions display dense PNNs surrounding the soma of neurons. Though their relative functions in individual brain regions and cell types may vary (Balmer, 2016; Bartos et al., 2002; Begum and Sng, 2017; Bernard and Prochiantz, 2016; Carstens et al., 2016; Dauth et al., 2016; de Winter et al., 2016; Deepa et al., 2002; Devienne et al., 2021; Dityatev et al., 2007; Donato et al., 2013; Durand et al., 2012; Frischknecht et al., 2009; Gundelfinger et al., 2010; Hartig et al., 1992; Hou et al., 2017; Kalemaki et al., 2018; Kosaka and Heizmann, 1989; Krishnan et al., 2017; Krishnan et al., 2015; Lau et al., 2020a; Liu et al., 2021; Nakagawa et al., 1986; Orlando et al., 2012; Pantazopoulos et al., 2020; Pizzorusso et al., 2006; Pizzorusso et al., 2002; Sigal et al., 2019; Sugiyama et al., 2009; Suttkus et al., 2012; Tansley et al., 2022; Tewari et al., 2018; Ueno et al., 2018; Vo et al., 2013; Wingert and Sorg, 2021; Ye and Miao, 2013), PNNs generally regulate the function of inhibitory networks during early postnatal development and across life. As PNNs are predominantly found on PV + GABAergic neurons in the cortex, they are intimately associated with, and contribute to, PV network function underlying cellular and organismal behavior (Agetsuma et al., 2018; Bartos et al., 2002; Cardin et al., 2009; Carstens et al., 2016; Cattaud et al., 2018; Cho et al., 2020; Donato et al., 2013; Faini et al., 2018; Gogolla et al., 2009; Karunakaran et al., 2016; Ko et al., 2013; Krishnan et al., 2017; Krishnan et al., 2015; Lau et al., 2020a; Miyata et al., 2012; Murthy et al., 2019; Nowicka et al., 2009; Sigal et al., 2019; Sohal et al., 2009; Thompson et al., 2018; Wingert and Sorg, 2021). Determining the function of PNNs with regional and biological contexts requires a thorough characterization of their expression across the whole brain (Lupori et al., 2023; Ueno et al., 2018). Particularly, Ueno et al. (2018) noted an increase in WFA + PNNs in the mouse V1B from two to 6 to 12 months of age, with no differences in V1M, from representative section analysis. This study highlights the need for systematic analysis across age and whole-brain regions. Here, we have performed a systematic analysis of PNN expression during the well-accepted critical period of the primary visual cortex (Figure 7).

In the early stages of visual cortex critical period development (~P15), WFA expression is generally diffuse in superficial layers, with minimal PNN structures in Layer IV as typically observed across visual cortex subregions (Figure 5). By the peak of critical period plasticity at P30, PNN expression is distinct in layers IV/V of V1B, compared to V1M and rostral V1 areas (Figures 2, 6). High expression of PNNs on parvalbumin+ GABAergic neurons in the primary sensory areas could selectively control thalamic excitation onto these neurons, and control of feed-forward thalamocortical sensory inputs into the sensory cortex (Faini et al., 2018; Lupori et al., 2023). Such functional connectivity regulations could have strong impact on the known roles of the binocular zone of the primary visual cortex in ocular dominance plasticity, binocular matching, and depth perception (Antonini et al., 1999; Fagiolini and Hensch, 2000; Krishnan et al., 2015; Pizzorusso et al., 2006; Pizzorusso et al., 2002; Wang et al., 2010). However, the functional relevance of PNN expression in V1M and rostral V1 requires further elucidation.

Typically, neurons of monocular V1 are thought to encode for receptive field properties including orientation and direction selectivity. However, the role of neuronal activity and/or visual experience in initial formation and maturation of these properties remains controversial (Chapman and Stryker, 1993; Hagihara et al., 2015; Ko et al., 2013; Miller et al., 1999). Furthermore, the role of inhibitory neurons in the establishment and maintenance of receptive field properties in monocular V1 remains to be determined. The emergence of multi-unit recordings and wide-field imaging techniques with natural scenery stimuli in different species could begin to shed light on the role of V1M and, ultimately, on parvalbumin+ interneurons and PNNs across subregions of the primary visual cortex.

As proteins involved in forming perineuronal nets are thought to be secreted by different cell types, the systematic coordination, assembly, and maintenance of PNNs during critical periods of development need further characterization. Cortical PNNs predominantly surround parvalbumin+ GABAergic neurons, the latter of which are typically identified through genetic strategies or immunostaining of parvalbumin protein. Though immunostaining is frequently used for quantitative analysis of parvalbumin expression, parvalbumin protein level is regulated in an activity-dependent manner (Berardi et al., 1993; Filice et al., 2016; Krishnan et al., 2015; Patz et al., 2004; Tropea et al., 2006; Ye and Miao, 2013). Thus, caution must be used in interpreting correlations between parvalbumin expression and PNN expression when solely relying on immunostaining techniques.

Neurodevelopmental disorders are synaptic disorders, which are particularly affected in early sensory critical periods when the fully formed brain is being sculpted by experience-driven neural activity. Studies in animal models of neurodevelopmental disorders such as Rett syndrome, Fragile X syndrome, and CDKL5 deficiency disorder have shown precocious or delayed impacts on sensory critical period processes, using both electrophysiological approaches and/or detection of altered PNN expression by immunostaining analysis (Chao et al., 2010; Durand et al., 2012; Han et al., 2012; Hensch, 2005; Hensch et al., 1998; Huang et al., 1999; Krishnan et al., 2015; Lupori et al., 2019; Mazziotti et al., 2017; Picard and Fagiolini, 2019; Pizzo et al., 2016; Terzic et al., 2021; Wen et al., 2018). Here, we present the first systematic characterization of PNN expression in the male mouse model of CDKL5 deficiency disorder with subregion-, anatomical-, and hemisphere-specificity during visual cortex critical period (Figure 7).

Our findings within the *Cdkl5*-null male primary visual cortex show both areas of agreement and divergence with Pizzo et al. (2016), who characterized PNN expression and PV + interneuron density in the V1B of *Cdkl5*-null mice at P18, P35, and in 8-week-old adults. In that study, adult null mice displayed increased PNN density, with no change in WFA staining intensity. Our P30 data from V1B are broadly consistent with this finding: we observe increased high-intensity PNN density and an increased proportion of high-intensity PNNs relative to all-PNNs in null V1B (Figures 3E,H, respectively), indicative of accelerated PNN maturation. However, our subregion-specific analysis reveals that this pattern is not uniform across the visual cortex: V1M shows a reduction in all-PNN density in null mice (Figure 3D), a divergence that would be masked in analyses restricted to V1B alone.

Pizzo et al. (2016) also reported that the age-dependent maturation of PNNs—reflected in a progressive shift from faint to strong WFA staining between P18 and P35—was completely abolished in null mice, with PNN staining remaining significantly fainter at P35 despite increased PNN numbers. Our findings suggest a more nuanced developmental trajectory. At P15, pixel-intensity analysis reveals broadly increased WFA signal in superficial and deep cortical layers across all V1 subregions in *Cdkl5*-null mice (Figure 5), representing diffuse extracellular matrix deposition that has not yet coalesced into identifiable PNN structures. By P30, this diffuse signal has resolved into region-specific patterns: V1B shows increased high-intensity PNN density and mean WFA intensity in null mice (Figures 3, 6), consistent with accelerated PNN maturation, while V1M shows reduced all-PNN density by manual counting despite increased pixel intensity in specific bands (Figures 3D, 6C–D). This apparent divergence between pixel-intensity and manual counting measures in V1M suggests that while extracellular matrix deposition may be elevated, the organization of this material into morphologically identifiable PNN structures follows a different trajectory in V1M compared to V1B. Extended analysis in V1B, V1M and rostral V1 over critical period also point to the earliest changes in dysregulated WFA expression at P15, indicating precocious atypical PNN expression in the *Cdkl5*-null male visual cortex (Figure 7A).

These subregion-specific developmental trajectories indicate that CDKL5 loss does not uniformly accelerate PNN maturation across the visual cortex but rather produces region-dependent effects that may reflect differences in the underlying inhibitory circuit development between V1 subregions.

Particularly, the consistent and early changes in the V1M and V1B are novel results, with implications for the imbalance between excitation and inhibition in the *Cdkl5*-null visual cortex. This finding aligns well with previously reported precocious critical period closure in the *Mecp2*-null visual cortex, which led to immature binocular matching patterns (Krishnan et al., 2015), and depth perception issues (Durand et al., 2012). Such atypical inhibitory changes to the neural network are likely to contribute to the visual impairments associated with CDKL5-deficiency disorder pathology. Further electrophysiological studies are necessary to determine how subregion-specific PNN expression differences (Figures 3, 6) ultimately contribute to the differentially evoked activity, *in vivo* neural activity and/or synchrony in the *Cdkl5*-null visual cortex across development.

Subregional differences to nuanced and likely compensatory synaptic plasticity mechanisms in the *Cdkl5*-null brain. Additionally, as parvalbumin+ interneuron networks influence PNN maintenance, it is likely that this network activity and/or gamma oscillations are disrupted in a subregion-specific manner in the *Cdkl5*-null visual cortex (Bartos et al., 2002; Devienne et al., 2021). Future work should determine the impact of classical critical period manipulations (e.g.: monocular deprivation, dark rearing) on PNN expression to determine the role of CDKL5 protein during this sensitive period. Conditional knockout of *Cdkl5* in glutamatergic neurons produces robust neurological phenotypes, indicative of a more significant role for CDKL5 in select neuronal subtypes over others (Awad et al., 2023; Lupori et al., 2019; Schroeder et al., 2019). From a translational or therapeutic perspective, exciting new biomarkers and candidates have been proposed in recent works (Saby et al., 2022; Van Bergen et al., 2022). However, as most of the above studies focus predominantly on null male mice (*including the present study*), further in-depth analyses across age are required using the female *Cdkl5*-heterozygous mouse model (Fuchs et al., 2018). Due to the subtle PNN phenotypes detected in null male mice, we could not justify performing similar visual cortical analysis in the *Cdkl5*-heterozygous female mice, due to the inherent mosaicism and lack of reliable antibodies to label CDKL5 protein in individual cells of the heterozygous females (unpublished data from the lab).

Previously, we reported that the adult female primary somatosensory cortex exhibited lateral-medial axis and hemisphere-specific changes in high-intensity PNN density, in both WT and *Mecp2*-heterozygous mice (Lau et al., 2020b). Such nuanced discovery was possible due to systematic whole brain analysis. We speculated that such anatomical differences could contribute to, or be shaped by, the functional specialization of the cortex. This functional specialization in the barrel cortex could contribute to the individual mouse’s whisker side preference, similar to handedness in primates (Lonsdorf and Hopkins, 2005). Determining when and how PNN lateralization occurs and is maintained across cortical subregions will require much work and seed new directions for the field. Toward this goal, we recently identified that adolescent 6-week-old female WT and *Mecp2*-heterozygous do not exhibit hemisphere laterality in PNN expression within the primary somatosensory cortex (Mykins et al., 2023), suggesting asymmetry in PNN density in the adult primary somatosensory cortex is an age-related, and likely experience-dependent, cellular phenotype. During these adolescent and early adulthood phases, tactile sensory perception is likely undergoing a sensitive period, influenced by puberty and gonadal hormones. Thus, it is likely that the maturation of PNN density and lateralization in expression in this cortical subregion is not finalized yet. Since the visual critical period matures earlier, we hypothesized an earlier lateralization of PNN expression in the primary visual cortex, though the functional relevance of such lateralization in rodents is unclear. Humans, on the other hand, are known to have a “dominant” eye (Chan and Chang, 2022; Dieter et al., 2017; Ooi et al., 2013; Ooi and He, 2020; Yang et al., 2010). However, we did not observe hemisphere or anatomical axes specific laterality in PNN density in the mouse primary visual cortex at P15 or P30. Additionally, this lack of lateralization was preserved in the *Cdkl5*-null male mice, indicating that CDKL5 is not required for establishing or maintaining this lateralization in developing male mice.

## Data Availability

The raw data supporting the conclusions of this article will be made available by the authors upon request.

## Footnotes

1
https://www.dx.doi.org/10.17504/protocols.io.bcf8itrw

## Supplementary material

Supplementary Figure 1 CDKL5-null male mice exhibit altered PNN expression across rostral-caudal axis in specific subregions of primary visual cortex at P30. **(A–F)** Distribution of all-PNN density **(A–C)** and high-intensity PNN density **(D–F)** in rostral V1 **(A, D)**, V1M **(B, E)** and V1B **(C, F)** of WT (black) and null (blue) (solid lines) across rostral-caudal axis. *N* = 1-13 images per Bregma coordinate, 5 animals per subregion. Bregma coordinates are based on Paxinos and Franklin, 2013. Solid lines + shades represent mean ± S.E.M.

## References

[ref1] Agetsuma M. Hamm J. P. Tao K. Fujisawa S. Yuste R. (2018). Parvalbumin-positive interneurons regulate neuronal ensembles in visual cortex. Cereb. Cortex 28, 1831–1845. doi: 10.1093/cercor/bhx169, 29106504 PMC5907345

[ref2] Antonini A. Fagiolini M. Stryker M. P. (1999). Anatomical correlates of functional plasticity in mouse visual cortex. J. Neurosci. 19, 4388–4406. doi: 10.1523/JNEUROSCI.19-11-04388.1999, 10341241 PMC2452998

[ref3] Awad P. N. Zerbi V. Johnson-Venkatesh E. M. Damiani F. Pagani M. Markicevic M. . (2023). CDKL5 sculpts functional callosal connectivity to promote cognitive flexibility. Mol. Psychiatry 29, 1698–1709. doi: 10.1038/s41380-023-01962-y, 36737483 PMC11371650

[ref4] Bahi-Buisson N. Bienvenu T. (2012). *CDKL5*-related disorders: from clinical description to molecular genetics. Mol. Syndromol. 2, 137–152. doi: 10.1159/000331333, 22670135 PMC3366705

[ref5] Bahi-Buisson N. Villeneuve N. Caietta E. Jacquette A. Maurey H. Matthijs G. . (2012). Recurrent mutations in the *CDKL5* gene: genotype-phenotype relationships. Am. J. Med. Genet. A 158A, 1612–1619. doi: 10.1002/ajmg.a.35401, 22678952

[ref6] Balmer T. S. (2016). Perineuronal nets enhance the excitability of fast-spiking neurons. eNeuro 3:112. doi: 10.1523/ENEURO.0112-16.2016, 27570824 PMC4987413

[ref7] Bartos M. Vida I. Frotscher M. Meyer A. Monyer H. Geiger J. R. P. . (2002). Fast synaptic inhibition promotes synchronized gamma oscillations in hippocampal interneuron networks. Proc. Natl. Acad. Sci. USA 99, 13222–13227. doi: 10.1073/pnas.192233099, 12235359 PMC130614

[ref8] Bavelier D. Levi D. M. Li R. W. Dan Y. Hensch T. K. (2010). Removing brakes on adult brain plasticity: from molecular to behavioral interventions. J. Neurosci. 30, 14964–14971. doi: 10.1523/JNEUROSCI.4812-10.2010, 21068299 PMC2992973

[ref9] Begum M. R. Sng J. C. G. (2017). Molecular mechanisms of experience-dependent maturation in cortical GABAergic inhibition. J. Neurochem. 142, 649–661. doi: 10.1111/jnc.14103, 28628196 PMC5599941

[ref10] Berardi N. Domenici L. Parisi V. Pizzorusso T. Cellerino A. Maffei L. (1993). Monocular deprivation effects in the rat visual cortex and lateral geniculate nucleus are prevented by nerve growth factor (NGF). I. Visual cortex. Proc. Biol. Sci. 251, 17–23. doi: 10.1098/rspb.1993.0003, 8094561

[ref11] Bernard C. Prochiantz A. (2016). Otx2-PNN interaction to regulate cortical plasticity. Neural Plast. 2016, 1–7. doi: 10.1155/2016/7931693, 26881132 PMC4736602

[ref12] Bignami A. Asher R. Perides G. (1992). Co-localization of hyaluronic acid and chondroitin sulfate proteoglycan in rat cerebral cortex. Brain Res. 579, 173–177. doi: 10.1016/0006-8993(92)90759-3, 1623404

[ref13] Brock D. Fidell A. Thomas J. Juarez-Colunga E. Benke T. A. Demarest S. (2021). Cerebral visual impairment in CDKL5 deficiency disorder correlates with developmental achievement. J. Child Neurol. 36, 974–980. doi: 10.1177/08830738211019284, 34547934 PMC9853471

[ref14] Brückner G. Bringmann A. Köppe G. Härtig W. Brauer K. (1996). In vivo and in vitro labelling of perineuronal nets in rat brain. Brain Res. 720, 84–92. doi: 10.1016/0006-8993(96)00152-7, 8782900

[ref15] Cardin J. A. Carlén M. Meletis K. Knoblich U. Zhang F. Deisseroth K. . (2009). Driving fast-spiking cells induces gamma rhythm and controls sensory responses. Nature 459, 663–667. doi: 10.1038/nature08002, 19396156 PMC3655711

[ref16] Carstens K. E. Lustberg D. J. Shaughnessy E. K. McCann K. E. Alexander G. M. Dudek S. M. (2021). Perineuronal net degradation rescues CA2 plasticity in a mouse model of Rett syndrome. J. Clin. Invest. 131:221. doi: 10.1172/JCI137221, 34228646 PMC8363283

[ref17] Carstens K. E. Phillips M. L. Pozzo-Miller L. Weinberg R. J. Dudek S. M. (2016). Perineuronal nets suppress plasticity of excitatory synapses on CA2 pyramidal neurons. J. Neurosci. 36, 6312–6320. doi: 10.1523/JNEUROSCI.0245-16.2016, 27277807 PMC4899529

[ref18] Carulli D. Rhodes K. E. Fawcett J. W. (2007). Upregulation of aggrecan, link protein 1, and hyaluronan synthases during formation of perineuronal nets in the rat cerebellum. J. Comp. Neurol. 501, 83–94. doi: 10.1002/cne.21231, 17206619

[ref19] Cattaud V. Bezzina C. Rey C. C. Lejards C. Dahan L. Verret L. (2018). Early disruption of parvalbumin expression and perineuronal nets in the hippocampus of the Tg2576 mouse model of Alzheimer’s disease can be rescued by enriched environment. Neurobiol. Aging 72, 147–158. doi: 10.1016/j.neurobiolaging.2018.08.024, 30273829

[ref20] Celio M. R. (1993). Perineuronal nets of extracellular matrix around parvalbumin-containing neurons of the hippocampus. Hippocampus 3, 55–60. 8287112

[ref21] Chan A. Y. C. Chang D. H. F. (2022). Neural correlates of sensory eye dominance in human visual white matter tracts. eneuro 9:ENEURO.0232-22.2022. doi: 10.1523/ENEURO.0232-22.2022, 36347601 PMC9698723

[ref22] Chao H.-T. Chen H. Samaco R. C. Xue M. Chahrour M. Yoo J. . (2010). Dysfunction in GABA signalling mediates autism-like stereotypies and Rett syndrome phenotypes. Nature 468, 263–269. doi: 10.1038/nature09582, 21068835 PMC3057962

[ref23] Chapman B. Stryker M. (1993). Development of orientation selectivity in ferret visual cortex and effects of deprivation. J. Neurosci. 13, 5251–5262. doi: 10.1523/JNEUROSCI.13-12-05251.1993, 8254372 PMC6576418

[ref001] Chavez M. C. Tremblay J. T. Zajkowski M. Ragusa M. Jones M. M. Whaley A. R. . (2026). Prenatal stress history modifies adolescent stress effects on adult social behavior and basolateral amygdala GABAergic neurons with perineuronal nets. Brain Research, 1882:150264. doi: 10.1016/j.brainres.2026.150264 41839325 PMC13179115

[ref24] Cho K. K. A. Davidson T. J. Bouvier G. Marshall J. D. Schnitzer M. J. Sohal V. S. (2020). Cross-hemispheric gamma synchrony between prefrontal parvalbumin interneurons supports behavioral adaptation during rule shift learning. Nat. Neurosci. 23, 892–902. doi: 10.1038/s41593-020-0647-1, 32451483 PMC7347248

[ref25] Dauth S. Grevesse T. Pantazopoulos H. Campbell P. H. Maoz B. M. Berretta S. . (2016). Extracellular matrix protein expression is brain region dependent. J. Comp. Neurol. 524, 1309–1336. doi: 10.1002/cne.23965, 26780384 PMC7714387

[ref26] de Winter F. Kwok J. C. F. Fawcett J. W. Vo T. T. Carulli D. Verhaagen J. (2016). The Chemorepulsive protein Semaphorin 3A and Perineuronal net-mediated plasticity. Neural Plast. 2016, 1–14. doi: 10.1155/2016/3679545, 27057361 PMC4738953

[ref27] Deepa S. S. Umehara Y. Higashiyama S. Itoh N. Sugahara K. (2002). Specific molecular interactions of oversulfated chondroitin sulfate E with various heparin-binding growth factors. Implications as a physiological binding partner in the brain and other tissues. J. Biol. Chem. 277, 43707–43716. doi: 10.1074/jbc.M207105200, 12221095

[ref28] Demarest S. Pestana-Knight E. M. Olson H. E. Downs J. Marsh E. D. Kaufmann W. E. . (2019). Severity assessment in CDKL5 deficiency disorder. Pediatr. Neurol. 97, 38–42. doi: 10.1016/j.pediatrneurol.2019.03.017, 31147226 PMC6659999

[ref29] Devienne G. Picaud S. Cohen I. Piquet J. Tricoire L. Testa D. . (2021). Regulation of perineuronal nets in the adult cortex by the activity of the cortical network. J. Neurosci. 41:434. doi: 10.1523/JNEUROSCI.0434-21.2021, 34045309 PMC8265812

[ref30] Dieter K. C. Sy J. L. Blake R. (2017). Individual differences in sensory eye dominance reflected in the dynamics of binocular rivalry. Vis. Res. 141, 40–50. doi: 10.1016/j.visres.2016.09.014, 27756700 PMC5406270

[ref31] Dityatev A. Brückner G. Dityateva G. Grosche J. Kleene R. Schachner M. (2007). Activity-dependent formation and functions of chondroitin sulfate-rich extracellular matrix of perineuronal nets. Dev. Neurobiol. 67, 570–588. doi: 10.1002/dneu.20361, 17443809

[ref32] Donato F. Rompani S. B. Caroni P. (2013). Parvalbumin-expressing basket-cell network plasticity induced by experience regulates adult learning. Nature 504, 272–276. doi: 10.1038/nature12866, 24336286

[ref33] Durand S. Patrizi A. Quast K. B. Hachigian L. Pavlyuk R. Saxena A. . (2012). NMDA receptor regulation prevents regression of visual cortical function in the absence of Mecp2. Neuron 76, 1078–1090. doi: 10.1016/j.neuron.2012.12.004, 23259945 PMC3733788

[ref34] Fagiolini M. Hensch T. K. (2000). Inhibitory threshold for critical-period activation in primary visual cortex. Nature 404, 183–186. doi: 10.1038/35004582, 10724170

[ref35] Faini G. Aguirre A. Landi S. Lamers D. Pizzorusso T. Ratto G. M. . (2018). Perineuronal nets control visual input via thalamic recruitment of cortical PV interneurons. eLife 7:e41520. doi: 10.7554/eLife.41520, 30561327 PMC6298774

[ref36] Fehr S. Downs J. Ho G. De Klerk N. Forbes D. Christodoulou J. . (2016). Functional abilities in children and adults with the CDKL5 disorder. Am. J. Med. Genet. A 170, 2860–2869. doi: 10.1002/ajmg.a.37851, 27528505

[ref37] Fehr S. Wilson M. Downs J. Williams S. Murgia A. Sartori S. . (2013). The CDKL5 disorder is an independent clinical entity associated with early-onset encephalopathy. Eur. J. Hum. Genet. 21, 266–273. doi: 10.1038/ejhg.2012.156, 22872100 PMC3573195

[ref38] Filice F. Vörckel K. J. Sungur A. Ö. Wöhr M. Schwaller B. (2016). Reduction in parvalbumin expression not loss of the parvalbumin-expressing GABA interneuron subpopulation in genetic parvalbumin and shank mouse models of autism. Mol. Brain 9:10. doi: 10.1186/s13041-016-0192-8, 26819149 PMC4729132

[ref39] Frischknecht R. Heine M. Perrais D. Seidenbecher C. I. Choquet D. Gundelfinger E. D. (2009). Brain extracellular matrix affects AMPA receptor lateral mobility and short-term synaptic plasticity. Nat. Neurosci. 12, 897–904. doi: 10.1038/nn.2338, 19483686

[ref40] Fuchs C. Gennaccaro L. Trazzi S. Bastianini S. Bettini S. Martire V. L. . (2018). Heterozygous CDKL5 knockout female mice are a valuable animal model for CDKL5 disorder. Neural Plast. 2018:e9726950. doi: 10.1155/2018/9726950, 29977282 PMC5994305

[ref41] Gogolla N. Caroni P. Lüthi A. Herry C. (2009). Perineuronal nets protect fear memories from erasure. Science 325, 1258–1261. doi: 10.1126/science.1174146, 19729657

[ref42] Guerrini R. Parrini E. (2012). Epilepsy in Rett syndrome, and CDKL5 - and FOXG1 -gene-related encephalopathies: MECP2-CDKL5-FOXG1- related encephalopathies. Epilepsia 53, 2067–2078. doi: 10.1111/j.1528-1167.2012.03656.x 22998673

[ref43] Gundelfinger E. D. Frischknecht R. Choquet D. Heine M. (2010). Converting juvenile into adult plasticity: a role for the brain’s extracellular matrix. Eur. J. Neurosci. 31, 2156–2165. doi: 10.1111/j.1460-9568.2010.07253.x, 20497467

[ref44] Hagihara K. M. Murakami T. Yoshida T. Tagawa Y. Ohki K. (2015). Neuronal activity is not required for the initial formation and maturation of visual selectivity. Nat. Neurosci. 18, 1780–1788. doi: 10.1038/nn.4155, 26523644

[ref45] Han S. Tai C. Westenbroek R. E. Yu F. H. Cheah C. S. Potter G. B. . (2012). Autistic-like behaviour in Scn1a+/− mice and rescue by enhanced GABA-mediated neurotransmission. Nature 489, 385–390. doi: 10.1038/nature11356, 22914087 PMC3448848

[ref46] Hartig W. Brauer K. Bruckner G. (1992). *Wisteria floribunda* agglutinin-labelled nets surround parvalbumin-containing neurons. Neuroreport 3, 869–872. doi: 10.1097/00001756-199210000-00012, 1421090

[ref47] Hensch T. K. (2005). Critical period mechanisms in developing visual cortex. Curr. Top. Dev. Biol. 69, 215–237. doi: 10.1016/s0070-2153(05)69008-4 16243601

[ref48] Hensch T. K. Gordon J. A. Brandon E. P. McKnight G. S. Idzerda R. L. Stryker M. P. (1998). Comparison of plasticity in vivo and in vitro in the developing visual cortex of Normal and protein kinase a RIβ-deficient mice. J. Neurosci. 18, 2108–2117. doi: 10.1523/JNEUROSCI.18-06-02108.1998, 9482797 PMC2553093

[ref49] Hou X. Yoshioka N. Tsukano H. Sakai A. Miyata S. Watanabe Y. . (2017). Chondroitin sulfate is required for onset and offset of critical period plasticity in visual cortex. Sci. Rep. 7:12646. doi: 10.1038/s41598-017-04007-x, 28974755 PMC5626782

[ref50] Huang Z. J. Kirkwood A. Pizzorusso T. Porciatti V. Morales B. Bear M. F. . (1999). BDNF regulates the maturation of inhibition and the critical period of plasticity in mouse visual cortex. Cell 98, 739–755. doi: 10.1016/s0092-8674(00)81509-3, 10499792

[ref51] Kalemaki K. Konstantoudaki X. Tivodar S. Sidiropoulou K. Karagogeos D. (2018). Mice with decreased number of interneurons exhibit aberrant spontaneous and oscillatory activity in the cortex. Front. Neural Circuits 12:96. doi: 10.3389/fncir.2018.00096, 30429776 PMC6220423

[ref52] Kalscheuer V. M. Tao J. Donnelly A. Hollway G. Schwinger E. Kübart S. . (2003). Disruption of the serine/threonine kinase 9 gene causes severe X-linked infantile spasms and mental retardation. Am. J. Hum. Genet. 72, 1401–1411. doi: 10.1086/375538, 12736870 PMC1180301

[ref53] Karunakaran S. Chowdhury A. Donato F. Quairiaux C. Michel C. M. Caroni P. (2016). PV plasticity sustained through D1/5 dopamine signaling required for long-term memory consolidation. Nat. Neurosci. 19, 454–464. doi: 10.1038/nn.4231, 26807952

[ref54] Ko H. Cossell L. Baragli C. Antolik J. Clopath C. Hofer S. B. . (2013). The emergence of functional microcircuits in visual cortex. Nature 496, 96–100. doi: 10.1038/nature12015, 23552948 PMC4843961

[ref55] Kosaka T. Heizmann C. W. (1989). Selective staining of a population of parvalbumin-containing GABAergic neurons in the rat cerebral cortex by lectins with specific affinity for terminal N-acetylgalactosamine. Brain Res. 483, 158–163. doi: 10.1016/0006-8993(89)90048-6, 2565147

[ref56] Krishnan K. Lau B. Y. B. Ewall G. Huang Z. J. Shea S. D. (2017). MECP2 regulates cortical plasticity underlying a learned behaviour in adult female mice. Nat. Commun. 8:14077. doi: 10.1038/ncomms14077, 28098153 PMC5253927

[ref57] Krishnan K. Wang B.-S. Lu J. Wang L. Maffei A. Cang J. . (2015). MeCP2 regulates the timing of critical period plasticity that shapes functional connectivity in primary visual cortex. Proc. Natl. Acad. Sci. USA 112, E4782–E4791. doi: 10.1073/pnas.1506499112, 26261347 PMC4553776

[ref58] Kwok J. C. F. Carulli D. Fawcett J. W. (2010). In vitro modeling of perineuronal nets: hyaluronan synthase and link protein are necessary for their formation and integrity: Hyaluronan synthase and link protein in PNNs. J. Neurochem. 114, 1447–1459. doi: 10.1111/j.1471-4159.2010.06878.x 20584105

[ref59] Lau B. Y. B. Krishnan K. Huang Z. J. Shea S. D. (2020a). Maternal experience-dependent cortical plasticity in mice is circuit- and stimulus-specific and requires MECP2. J. Neurosci. 40, 1514–1526. doi: 10.1523/JNEUROSCI.1964-19.2019, 31911459 PMC7044728

[ref60] Lau B. Y. B. Layo D. E. Emery B. Everett M. Kumar A. Stevenson P. . (2020b). Lateralized expression of cortical perineuronal nets during maternal experience is dependent on MECP2. eNeuro 7:ENEURO.0500-19.2020. doi: 10.1523/ENEURO.0500-19.2020, 32332080 PMC7294466

[ref61] LeBlanc J. J. DeGregorio G. Centofante E. Vogel-Farley V. K. Barnes K. Kaufmann W. E. . (2015). Visual evoked potentials detect cortical processing deficits in Rett syndrome: VEP in Rett syndrome. Ann. Neurol. 78, 775–786. doi: 10.1002/ana.24513, 26332183 PMC7374762

[ref62] Levelt C. N. Hübener M. (2012). Critical-period plasticity in the visual cortex. Annu. Rev. Neurosci. 35, 309–330. doi: 10.1146/annurev-neuro-061010-113813, 22462544

[ref63] Liu Y.-J. Spangenberg E. E. Tang B. Holmes T. C. Green K. N. Xu X. (2021). Microglia elimination increases neural circuit connectivity and activity in adult mouse cortex. J. Neurosci. 41, 1274–1287. doi: 10.1523/JNEUROSCI.2140-20.2020, 33380470 PMC7888230

[ref64] Lonsdorf E. V. Hopkins W. D. (2005). Wild chimpanzees show population-level handedness for tool use. Proc. Natl. Acad. Sci. 102, 12634–12638. doi: 10.1073/pnas.0505806102, 16105943 PMC1194943

[ref65] Lupori L. Sagona G. Fuchs C. Mazziotti R. Stefanov A. Putignano E. . (2019). Site-specific abnormalities in the visual system of a mouse model of CDKL5 deficiency disorder. Hum. Mol. Genet. 28, 2851–2861. doi: 10.1093/hmg/ddz102, 31108505 PMC6736061

[ref66] Lupori L. Totaro V. Cornuti S. Ciampi L. Carrara F. Grilli E. . (2023). A comprehensive atlas of perineuronal net distribution and colocalization with parvalbumin in the adult mouse brain. Cell Rep. 42:112788. doi: 10.1016/j.celrep.2023.112788 37436896

[ref67] Mari F. Azimonti S. Bertani I. Bolognese F. Colombo E. Caselli R. . (2005). CDKL5 belongs to the same molecular pathway of MeCP2 and it is responsible for the early-onset seizure variant of Rett syndrome. Hum. Mol. Genet. 14, 1935–1946. doi: 10.1093/hmg/ddi198, 15917271

[ref68] Mazziotti R. Lupori L. Sagona G. Gennaro M. Della Sala G. Putignano E. . (2017). Searching for biomarkers of CDKL5 disorder: early-onset visual impairment in CDKL5 mutant mice. Hum. Mol. Genet. 26, 2290–2298. doi: 10.1093/hmg/ddx119, 28369421 PMC5458338

[ref69] Miller K. D. Erwin E. Kayser A. (1999). Is the development of orientation selectivity instructed by activity? J. Neurobiol. 41, 44–57. doi: 10.1002/(SICI)1097-4695(199910)41:1<44::AID-NEU7>3.0.CO;2-V, 10504191

[ref70] Miyata S. Kitagawa H. (2017). Formation and remodeling of the brain extracellular matrix in neural plasticity: roles of chondroitin sulfate and hyaluronan. Biochim. Biophys. Acta 1861, 2420–2434. doi: 10.1016/j.bbagen.2017.06.010, 28625420

[ref71] Miyata S. Komatsu Y. Yoshimura Y. Taya C. Kitagawa H. (2012). Persistent cortical plasticity by upregulation of chondroitin 6-sulfation. Nat. Neurosci. 15, 414–422. doi: 10.1038/nn.3023, 22246436

[ref72] Murthy S. Kane G. A. Katchur N. J. Lara Mejia P. S. Obiofuma G. Buschman T. J. . (2019). Perineuronal nets, inhibitory interneurons, and anxiety-related ventral hippocampal neuronal oscillations are altered by early life adversity. Biol. Psychiatry 85, 1011–1020. doi: 10.1016/j.biopsych.2019.02.021, 31027646 PMC6590696

[ref73] Mykins M. Layo-Carris D. Dunn L. R. Skinner D. W. McBryar A. H. Perez S. . (2023). Wild-type MECP2 expression coincides with age-dependent sensory phenotypes in a female mouse model for Rett syndrome. J. Neurosci. Res. 101, 1236–1258. doi: 10.1002/jnr.25190, 37026482 PMC10332853

[ref74] Nakagawa F. Schulte B. A. Wu J. Y. Spicer S. S. (1986). GABAergic neurons of rodent brain correspond partially with those staining for glycoconjugate with terminal N-acetylgalactosamine. J. Neurocytol. 15, 389–396. doi: 10.1007/BF01611440, 2427661

[ref75] Nowicka D. Soulsby S. Skangiel-Kramska J. Glazewski S. (2009). Parvalbumin-containing neurons, perineuronal nets and experience-dependent plasticity in murine barrel cortex. Eur. J. Neurosci. 30, 2053–2063. doi: 10.1111/j.1460-9568.2009.06996.x, 20128844

[ref76] Ooi T. L. He Z. J. (2020). Sensory eye dominance: relationship between eye and brain. Eye Brain 12, 25–31. doi: 10.2147/EB.S176931, 32021530 PMC6980844

[ref77] Ooi T. L. Su Y. R. Natale D. M. He Z. J. (2013). A push-pull treatment for strengthening the ‘lazy eye’ in amblyopia. Curr. Biol. 23, R309–R310. doi: 10.1016/j.cub.2013.03.004, 23618663 PMC6485254

[ref78] Orlando C. Ster J. Gerber U. Fawcett J. W. Raineteau O. (2012). Perisynaptic chondroitin sulfate proteoglycans restrict structural plasticity in an integrin-dependent manner. J. Neurosci. 32, 18009–18017. doi: 10.1523/JNEUROSCI.2406-12.2012, 23238717 PMC6621736

[ref79] Pantazopoulos H. Gisabella B. Rexrode L. Benefield D. Yildiz E. Seltzer P. . (2020). Circadian rhythms of perineuronal net composition. eNeuro 7:2020. doi: 10.1523/ENEURO.0034-19.2020, 32719104 PMC7405073

[ref80] Patrizi A. Awad P. N. Chattopadhyaya B. Li C. Di Cristo G. Fagiolini M. (2020). Accelerated hyper-maturation of parvalbumin circuits in the absence of MeCP2. Cereb. Cortex 30, 256–268. doi: 10.1093/cercor/bhz085, 31038696 PMC7029683

[ref81] Patz S. Grabert J. Gorba T. Wirth M. J. Wahle P. (2004). Parvalbumin expression in visual cortical interneurons depends on neuronal activity and TrkB ligands during an early period of postnatal development. Cereb. Cortex 14, 342–351. doi: 10.1093/cercor/bhg132, 14754872

[ref82] Paxinos G. Franklin K. B. J. (2013). Paxino’s and Franklin’s The Mouse Brain In Stereotaxic Coordinates, 4th Ed. Fourth. Edn Waltham, MA: Academic Press.

[ref83] Picard N. Fagiolini M. (2019). MeCP2: an epigenetic regulator of critical periods. Curr. Opin. Neurobiol. 59, 95–101. doi: 10.1016/j.conb.2019.04.004, 31163286

[ref84] Pizzo R. Gurgone A. Castroflorio E. Amendola E. Gross C. Sassoè-Pognetto M. . (2016). Lack of Cdkl5 disrupts the Organization of Excitatory and Inhibitory Synapses and Parvalbumin interneurons in the primary visual cortex. Front. Cell. Neurosci. 10:261. doi: 10.3389/fncel.2016.00261, 27965538 PMC5124713

[ref85] Pizzorusso T. Medini P. Berardi N. Chierzi S. Fawcett J. W. Maffei L. (2002). Reactivation of ocular dominance plasticity in the adult visual cortex. Science 298, 1248–1251. doi: 10.1126/science.1072699, 12424383

[ref86] Pizzorusso T. Medini P. Landi S. Baldini S. Berardi N. Maffei L. (2006). Structural and functional recovery from early monocular deprivation in adult rats. Proc. Natl. Acad. Sci. 103, 8517–8522. doi: 10.1073/pnas.0602657103, 16709670 PMC1482523

[ref87] Quintiliani M. Ricci D. Petrianni M. Leone S. Orazi L. Amore F. . (2021). Cortical visual impairment in CDKL5 deficiency disorder. Front. Neurol. 12:805745. doi: 10.3389/fneur.2021.805745, 35153983 PMC8825365

[ref88] Reinhard S. M. Rais M. Afroz S. Hanania Y. Pendi K. Espinoza K. . (2019). Reduced perineuronal net expression in Fmr1 KO mice auditory cortex and amygdala is linked to impaired fear-associated memory. Neurobiol. Learn. Mem. 164:107042. doi: 10.1016/j.nlm.2019.107042, 31326533 PMC7519848

[ref89] Saby J. N. Mulcahey P. J. Zavez A. E. Peters S. U. Standridge S. M. Swanson L. C. . (2022). Electrophysiological biomarkers of brain function in CDKL5 deficiency disorder. Brain Commun. 4:fcac197. doi: 10.1093/braincomms/fcac197, 35974796 PMC9374482

[ref90] Schroeder E. Yuan L. Seong E. Ligon C. DeKorver N. Gurumurthy C. B. . (2019). Neuron-type specific loss of CDKL5 leads to alterations in mTOR signaling and synaptic markers. Mol. Neurobiol. 56, 4151–4162. doi: 10.1007/s12035-018-1346-8, 30288694 PMC6447488

[ref002] Sakata J. T. Alonge K. M. Diethorn E. J. Krishnan K. Milman N. E. P. Ramsaran A. I. . (2025). Critical Periods and Beyond: Dynamic Functions of Perineuronal Nets in Cognition, Development, and Disease. Journal of Neuroscience, 45, 1–13. doi: 10.1523/JNEUROSCI.1367-25.2025 PMC12614057 41224655

[ref91] Sigal Y. M. Bae H. Bogart L. J. Hensch T. K. Zhuang X. (2019). Structural maturation of cortical perineuronal nets and their perforating synapses revealed by superresolution imaging. Proc. Natl. Acad. Sci. USA 116, 7071–7076. doi: 10.1073/pnas.1817222116, 30890637 PMC6452715

[ref92] Siri B. Varesio C. Freri E. Darra F. Gana S. Mei D. . (2021). CDKL5 deficiency disorder in males: five new variants and review of the literature. Eur. J. Paediatr. Neurol. 33, 9–20. doi: 10.1016/j.ejpn.2021.04.007, 33989939

[ref93] Slaker M. L. Harkness J. H. Sorg B. A. (2016). A standardized and automated method of perineuronal net analysis using *Wisteria floribunda* agglutinin staining intensity. IBRO Rep. 1, 54–60. doi: 10.1016/j.ibror.2016.10.001, 28713865 PMC5507617

[ref94] Sohal V. S. Zhang F. Yizhar O. Deisseroth K. (2009). Parvalbumin neurons and gamma rhythms enhance cortical circuit performance. Nature 459, 698–702. doi: 10.1038/nature07991, 19396159 PMC3969859

[ref95] Sorg B. A. Berretta S. Blacktop J. M. Fawcett J. W. Kitagawa H. Kwok J. C. F. . (2016). Casting a wide net: role of perineuronal nets in neural plasticity. J. Neurosci. 36, 11459–11468. doi: 10.1523/JNEUROSCI.2351-16.2016, 27911749 PMC5125213

[ref96] Sugiyama S. Prochiantz A. Hensch T. K. (2009). From brain formation to plasticity: insights on Otx2 homeoprotein: Otx2 transfer shapes postnatal circuits. Develop. Growth Differ. 51, 369–377. doi: 10.1111/j.1440-169X.2009.01093.x 19298552

[ref97] Suttkus A. Rohn S. Jäger C. Arendt T. Morawski M. (2012). Neuroprotection against iron-induced cell death by perineuronal nets - an in vivo analysis of oxidative stress. Am. J. Neurodegener. Dis. 1, 122–129. 23383386 PMC3560462

[ref98] Takesian A. E. Hensch T. K. (2013). Balancing plasticity/stability across brain development. Prog. Brain Res. 22, 3–34. doi: 10.1016/B978-0-444-63327-9.00001-1 24309249

[ref99] Tansley S. Gu N. Guzmán A. U. Cai W. Wong C. Lister K. C. . (2022). Microglia-mediated degradation of perineuronal nets promotes pain. Science 377, 80–86. doi: 10.1126/science.abl6773, 35617374

[ref100] Terzic B. Davatolhagh M. F. Ho Y. Tang S. Liu Y.-T. Xia Z. . (2021). Temporal manipulation of Cdkl5 reveals essential postdevelopmental functions and reversible CDKL5 deficiency disorder-related deficits. J. Clin. Invest. 131:e143655. doi: 10.1172/JCI143655, 34651584 PMC8516470

[ref101] Tewari B. P. Chaunsali L. Campbell S. L. Patel D. C. Goode A. E. Sontheimer H. (2018). Perineuronal nets decrease membrane capacitance of peritumoral fast spiking interneurons in a model of epilepsy. Nat. Commun. 9:4724. doi: 10.1038/s41467-018-07113-0, 30413686 PMC6226462

[ref102] Thompson E. H. Lensjø K. K. Wigestrand M. B. Malthe-Sørenssen A. Hafting T. Fyhn M. (2018). Removal of perineuronal nets disrupts recall of a remote fear memory. Proc. Natl. Acad. Sci. USA 115, 607–612. doi: 10.1073/pnas.1713530115, 29279411 PMC5776974

[ref103] Tropea D. Kreiman G. Lyckman A. Mukherjee S. Yu H. Horng S. . (2006). Gene expression changes and molecular pathways mediating activity-dependent plasticity in visual cortex. Nat. Neurosci. 9, 660–668. doi: 10.1038/nn1689, 16633343

[ref104] Ueno H. Takao K. Suemitsu S. Murakami S. Kitamura N. Wani K. . (2018). Age-dependent and region-specific alteration of parvalbumin neurons and perineuronal nets in the mouse cerebral cortex. Neurochem. Int. 112, 59–70. doi: 10.1016/j.neuint.2017.11.001, 29126935

[ref105] Van Bergen N. J. Massey S. Quigley A. Rollo B. Harris A. R. Kapsa R. M. I. . (2022). CDKL5 deficiency disorder: molecular insights and mechanisms of pathogenicity to fast-track therapeutic development. Biochem. Soc. Trans. 50, 1207–1224. doi: 10.1042/BST20220791, 35997111 PMC9444073

[ref106] Vo T. Carulli D. Ehlert E. M. E. Kwok J. C. F. Dick G. Mecollari V. . (2013). The chemorepulsive axon guidance protein semaphorin3A is a constituent of perineuronal nets in the adult rodent brain. Mol. Cell. Neurosci. 56, 186–200. doi: 10.1016/j.mcn.2013.04.009, 23665579

[ref107] Wang I.-T. J. Allen M. Goffin D. Zhu X. Fairless A. H. Brodkin E. S. . (2012). Loss of CDKL5 disrupts kinome profile and event-related potentials leading to autistic-like phenotypes in mice. Proc. Natl. Acad. Sci. USA 109, 21516–21521. doi: 10.1073/pnas.1216988110, 23236174 PMC3535652

[ref108] Wang B.-S. Sarnaik R. Cang J. (2010). Critical period plasticity matches binocular orientation preference in the visual cortex. Neuron 65, 246–256. doi: 10.1016/j.neuron.2010.01.002, 20152130 PMC2822731

[ref109] Weaving L. S. Christodoulou J. Williamson S. L. Friend K. L. McKenzie O. L. D. Archer H. . (2004). Mutations of CDKL5 cause a severe neurodevelopmental disorder with infantile spasms and mental retardation. Am. J. Hum. Genet. 75, 1079–1093. doi: 10.1086/426462, 15492925 PMC1182143

[ref110] Wen T. H. Afroz S. Reinhard S. M. Palacios A. R. Tapia K. Binder D. K. . (2018). Genetic reduction of matrix Metalloproteinase-9 promotes formation of Perineuronal nets around Parvalbumin-expressing interneurons and normalizes auditory cortex responses in developing Fmr1 Knock-out mice. Cereb. Cortex 28, 3951–3964. doi: 10.1093/cercor/bhx258, 29040407 PMC6188540

[ref111] Wingert J. C. Sorg B. A. (2021). Impact of perineuronal nets on electrophysiology fo parvalbumin interneurons, principal neurons, and brain oscillations: a review. Front. Synaptic Neurosci. 10:673210. doi: 10.3389/fnsyn.2021.673210, 34040511 PMC8141737

[ref112] Xia D. Li L. Yang B. Zhou Q. (2021). Altered relationship between Parvalbumin and Perineuronal nets in an autism model. Front. Mol. Neurosci. 14:597812. doi: 10.3389/fnmol.2021.597812, 33912009 PMC8072465

[ref113] Yang E. Blake R. McDonald J. E. (2010). A new interocular suppression technique for measuring sensory eye dominance. Invest. Ophthalmol. Vis. Sci. 51:588. doi: 10.1167/iovs.08-3076, 19628736 PMC2810859

[ref114] Ye Q. Miao Q. (2013). Experience-dependent development of perineuronal nets and chondroitin sulfate proteoglycan receptors in mouse visual cortex. Matrix Biol. 32, 352–363. doi: 10.1016/j.matbio.2013.04.001, 23597636

[ref115] Zhu Y.-C. Xiong Z.-Q. (2019). Molecular and synaptic bases of CDKL5 disorder. Dev. Neurobiol. 79, 8–19. doi: 10.1002/Tdneu.22639 30246934

