# Supplementary figures and images for: CDKL5 deficiency results in atypical subregion-specific expression of perineuronal nets during mouse visual critical period
Source: Front Neurosci. 2026 May 20;20:1769195. doi: 10.3389/fnins.2026.1769195 (PMC13230037; doi:10.3389/fnins.2026.1769195)

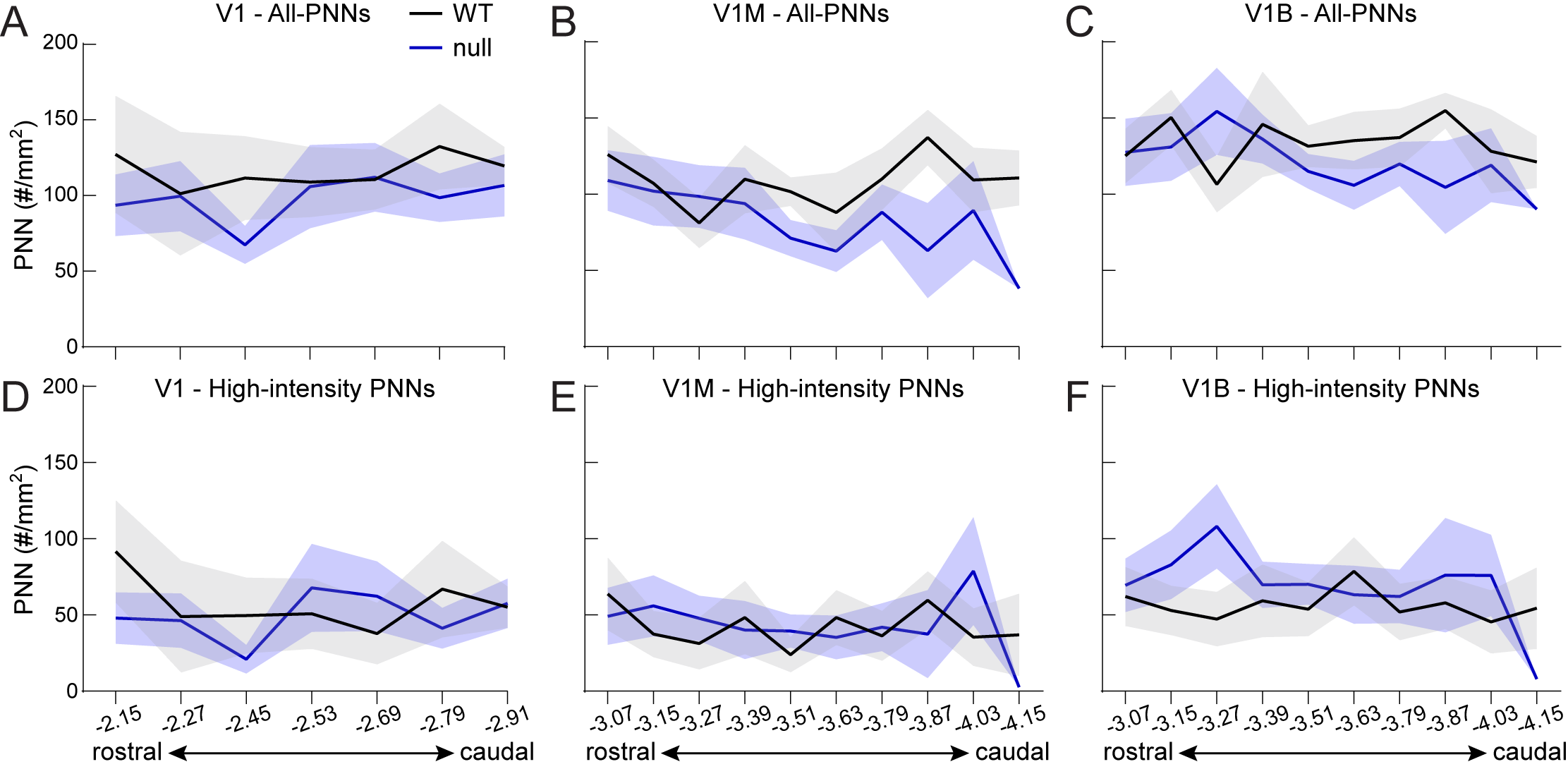

Supplement: Supplementary Figure 1 — CDKL5-null male mice exhibit altered PNN expression across rostral-caudal axis in specific subregions of primary visual cortex at P30. (A–F) Distribution of all-PNN density (A–C) and high-intensity PNN density (D–F) in rostral V1 (A, D), V1M (B, E) and V1B (C, F) of WT (black) and null (blue) (solid lines) across rostral-caudal axis. N = 1-13 images per Bregma coordinate, 5 animals per subregion. Bregma coordinates are based on Paxinos and Franklin, 2013. Solid lines + shades represent mean ± S.E.M. [file Image_1.tif]
